# LncRNA SNHG17 interacts with LRPPRC to stabilize c-Myc protein and promote G1/S transition and cell proliferation

**DOI:** 10.1038/s41419-021-04238-x

**Published:** 2021-10-20

**Authors:** Jin-Yu Liu, Ya-Jing Chen, Huan-Hui Feng, Zhan-Li Chen, Yun-Long Wang, Jin-E Yang, Shi-Mei Zhuang

**Affiliations:** 1 MOE Key Laboratory of Gene Function and Regulation, School of Life Sciences, Sun Yat-sen University, Xin Gang Xi Road 135#, Guangzhou, 510275 P. R. China; 2 Key Laboratory of Liver Disease of Guangdong Province, the Third Affiliated Hospital, Sun Yat-sen University, Guangzhou, China

**Keywords:** Oncogenes, Cell growth, Long non-coding RNAs

## Abstract

Oncogenic c-Myc is a master regulator of G1/S transition. Long non-coding RNAs (lncRNAs) emerge as new regulators of various cell activities. Here, we found that lncRNA SnoRNA Host Gene 17 (SNHG17) was elevated at the early G1-phase of cell cycle. Both gain- and loss-of function studies disclosed that SNHG17 increased c-Myc protein level, accelerated G1/S transition and cell proliferation, and consequently promoted tumor cell growth in vitro and in vivo. Mechanistically, the 1-150-nt of SNHG17 physically interacted with the 1035-1369-aa of leucine rich pentatricopeptide repeat containing (LRPPRC) protein, and disrupting this interaction abrogated the promoting role of SNHG17 in c-Myc expression, G1/S transition, and cell proliferation. The effect of SNHG17 in stimulating cell proliferation was attenuated by silencing c-Myc or LRPPRC. Furthermore, silencing SNHG17 or LRPPRC increased the level of ubiquitylated c-Myc and reduced the stability of c-Myc protein. Analysis of human hepatocellular carcinoma (HCC) tissues revealed that SNHG17, LRPPRC, and c-Myc were significantly upregulated in HCC, and they showed a positive correlation with each other. High level of SNHG17 or LRPPRC was associated with worse survival of HCC patients. These data suggest that SNHG17 may inhibit c-Myc ubiquitination and thus enhance c-Myc level and facilitate proliferation by interacting with LRPPRC. Our findings identify a novel SNHG17-LRPPRC-c-Myc regulatory axis and elucidate its roles in G1/S transition and tumor growth, which may provide potential targets for cancer therapy.

## Introduction

The G1 to S phase (G1/S) transition is the key step that drives cell to the division cycle, and it is tightly controlled by the cyclin-dependent kinase (CDK)-Rb-E2F pathway [1, 2]. c-Myc is a master regulator of cell proliferation via acting as a transcription factor. Upon stimulation of growth signals, c-Myc increases rapidly and then activates the transcription of CDKs, cyclins, E2F1, and/or inhibits the expression of CDK inhibitors, resulting in accelerating G1/S transition and cell proliferation [3, 4].

Upregulation of c-Myc is frequently observed in a variety of tumors. Abnormal overexpression of c-Myc facilitates cancer development and progression by promoting cell proliferation, metabolic adaptation and suppressing the antitumor immune response [4–7], suggesting c-Myc as an attractive target for anti-cancer therapy. However, targeting c-Myc directly is intractable owing to its lack of a hydrophobic pocket that is compatible with a small organic molecule [8]. Nevertheless, recent studies in animal models show that selective small molecules targeting the BET bromodomains-containing proteins that regulate c-Myc activity can downregulate the c-Myc transcriptional signaling, reduce tumor burden, and prolong survival. Some of these small molecules already enter Phase I clinical trials [9, 10]. Therefore, targeting c-Myc regulators may represent a potential strategy for anti-cancer therapy to inhibit c-Myc activity.

Long non-coding RNAs (lncRNAs) belong to a class of non-coding transcripts longer than 200 nucleotides [11]. They may work as signals, as scaffolds for protein–protein interactions, as molecular decoys, or guides to target elements [12]. LncRNAs have emerged as new regulators of various cell activities, such as cell proliferation, death, and differentiation [13, 14]. Deregulation of lncRNAs can promote tumor development, self-renewal of cancer stem cells, and drug resistance [15, 16]. Recent evidence shows that knockdown of lncRNA EPIC1 reduces the c-Myc’s occupancy on the promoters of c-Myc target genes and curtails their expression [17]. In contrast, lncRNA GLCC1 and PVT1 can increase the stability of c-Myc protein [18, 19]. These findings suggest that lncRNAs may play important roles in regulating c-Myc function. However, lncRNAs that regulate c-Myc and, in turn, cell cycle progression remain to be explored.

In an attempt to identify oncogenic lncRNAs that regulate G1/S transition, we screened for the lncRNAs that were differentially expressed during cell cycle progression and found that lncRNA SnoRNA Host Gene 17 (SNHG17) was upregulated at the early G1-phase and in various cancer types, including hepatocellular carcinoma (HCC). It increased the stability of c-Myc protein and in turn accelerated G1/S transition and cell proliferation via physically interacting with leucine rich pentatricopeptide repeat containing (LRPPRC) protein. These findings reveal the novel roles of SNHG17 and LRPPRC in regulating c-Myc function and highlight the importance of the SNHG17-LRPPRC-c-Myc axis in G1/S transition and tumor growth, which may provide potential targets for cancer therapy.

## Results

### SNHG17 is upregulated at the early G1-phase and increases the stability of c-Myc protein

**Fig. 1:**
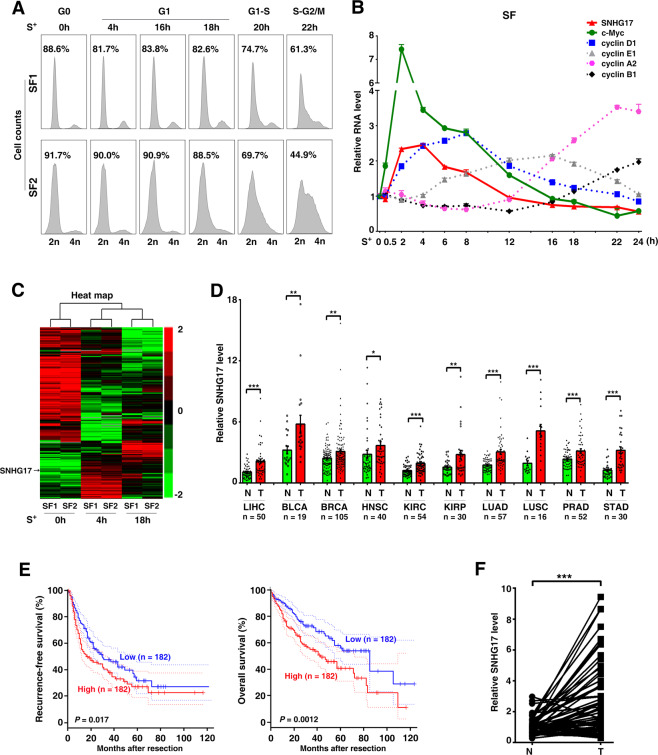
Screening for lncRNAs that may regulate G1/S transition. **A**–**B** The cell cycle distribution and the expression pattern of cell cycle-related genes in serum starvation-stimulation models. SF cells were serum-deprived and then cultured in the 15% FBS-containing medium at the indicated time periods before FACS (**A**) or qPCR (**B**). The percentage of cells at the G1-phase is indicated for each sample (**A**). The time point for serum re-addition was set as 0 h. **C** Heat map of lncRNA expression profiles during cell cycle progression. For **A**–**C**, S^+^ indicates serum re-addition. **D** Pan-cancer analysis of SNHG17 expression based on TCGA data. The levels of SNHG17 in paired tumor (T) and non-tumor (N) tissues from 10 different cancer types are presented. LIHC, liver hepatocellular carcinoma; BLCA, bladder urothelial carcinoma; BRCA, breast invasive carcinoma; HNSC, head and neck squamous cell carcinoma; KIRC, kidney renal clear cell carcinoma; KIRP, kidney renal papillary cell carcinoma; LUAD, lung adenocarcinoma; LUSC, lung squamous cell carcinoma; PRAD, prostate adenocarcinoma; STAD, stomach adenocarcinoma. **E** Kaplan–Meier analysis revealed a significant association between higher SNHG17 level and shorter recurrence-free survival or overall survival of HCC patients. The median SNHG17 level in all 364 HCC tissues, derived from TCGA, was chosen as the cut-off value to separate the high-SNHG17 group (*n* = 182) from the low-SNHG17 group (*n* = 182). **F** The level of SNHG17 was significantly increased in HCC tissues of our study cohort. The expression of SNHG17 was assessed by qPCR in 60 paired HCC (T) and adjacent non-tumor liver tissues (N). For **B**, error bars represent mean ± SEM from three independent experiments. *P* values were assessed by paired Student’s *t* test (**D**, **F**) or log-rank test (**E**). *, *P* < 0.05; **, P < 0.01; ***, P < 0.001.

**Fig. 2:**
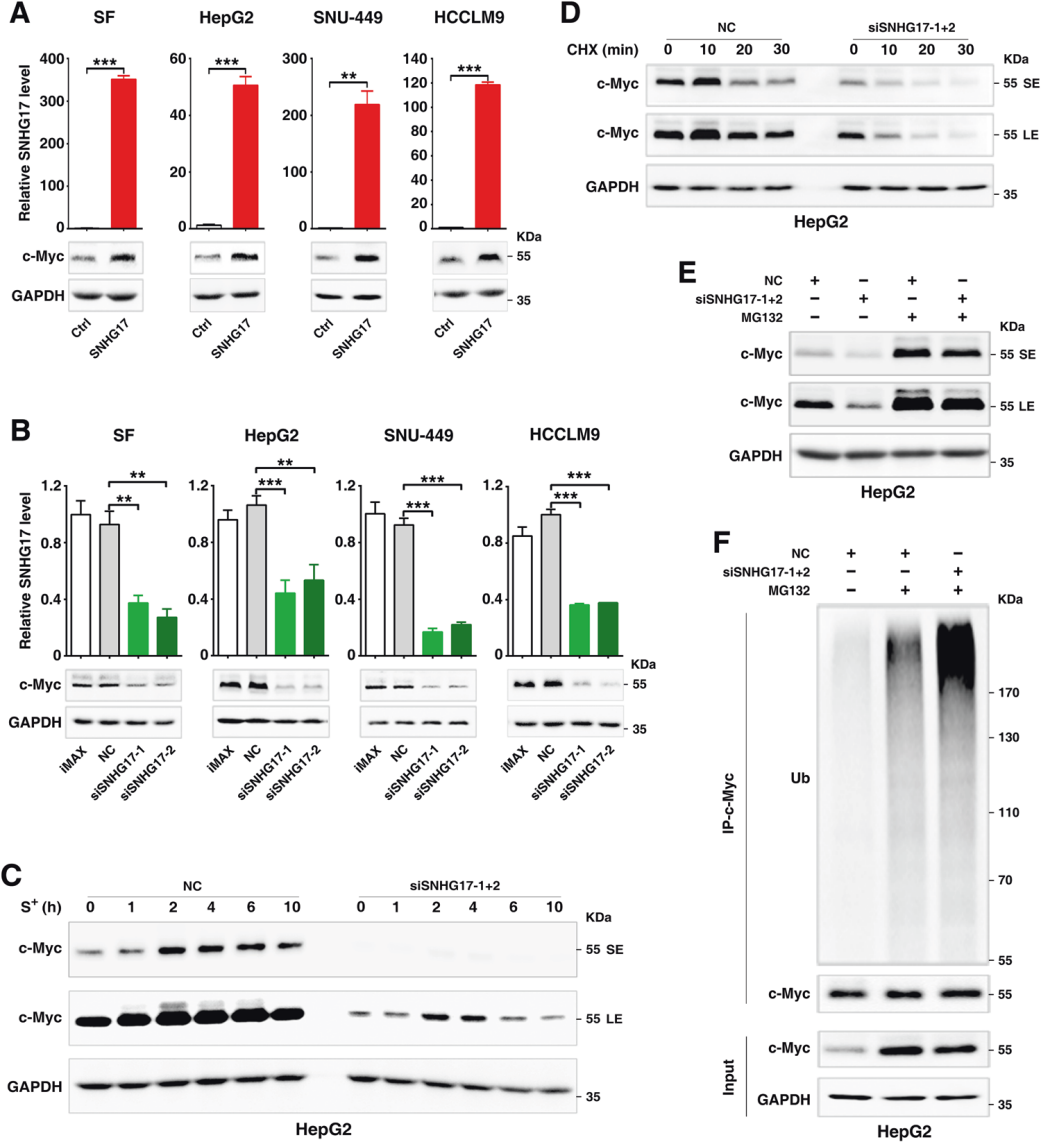
SNHG17 increases the stability of c-Myc protein. **A** Overexpression of SNHG17 increased the protein level of c-Myc. Cells stably expressing SNHG17 and the control cells (Ctrl) were analyzed by qPCR (up panel) and Western blotting (down panel). **B** Silencing SNHG17 decreased the protein level of c-Myc. NC- or siSNHG17-tranfected cells were subjected to qPCR analysis (up panel) and Western blotting (down panel). **C** Silencing SNHG17 accelerated the decline of c-Myc protein during cell cycle progression. NC- or siSNHG17-transfectants were serum-starved and then grown in the 15% FBS-containing medium (S^+^) for the indicated hours, followed by Western blotting. SE, short exposure; LE, long exposure. **D** Silencing SNHG17 decreased the stability of c-Myc protein. NC- or siSNHG17-transfected cells were treated with 25 μg/mL cycloheximide for the indicated time periods before Western blotting. **E** The siSNHG17-induced c-Myc reduction was attenuated by proteasome inhibitor. NC- or siSNHG17-transfectants without (−) or with (+) 10 μg/mL MG132 treatment for 3 h were subjected to Western blotting. **F** Silencing SNHG17 increased the level of ubiquitinated c-Myc. NC- or siSNHG17-transfectants without (−) or with (+) 10 μg/mL MG132 treatment for 3 h were subjected to immunoprecipitation (IP) using anti-c-Myc antibody and then Western blotting using anti-Ubiquitin antibody (Ub). iMAX, cells exposed to Lipofectamine RNAiMAX but not RNA duplexes. NC, negative control of RNA duplex. siSNHG17-1 and siSNHG17-2, siRNAs targeting different regions of SNHG17. Different exposure times were used to achieve appropriate signal-to-noise for different experiments. For **A**–**B**, error bars represent mean ± SEM from three independent experiments. *P* values were assessed by unpaired Student’s *t* test. **, *P* < 0.01; ***, P < 0.001.

To identify oncogenic lncRNAs that may regulate G1/S transition, we adopted RNA sequencing technology to screen for molecules that were differentially expressed during cell cycle progression, using a serum starvation-stimulation assay in human skin fibroblasts (SF), a classic model for G1/S transition. The expression pattern of cell cycle-related genes indicated the successful establishment of the studied model (Fig. 1A–B). As shown, SF cells were induced to enter a quiescent state by depriving serum for 48 h and then stimulated to enter the G1-phase and subsequent S-phase by adding serum (Fig. 1A). Next, we examined the expression profiling of genes at 0, 4, and 18 h after serum addition, representing G0, early, and late G1-phase, respectively. We found that 511 lncRNAs were differentially expressed at 4 or 18 h compared to 0 h after serum stimulation (Fig. 1C). The following criteria were then used to select the candidate lncRNAs (Supplementary Fig. S1A): (1) Location in intergenic regions of human genome; (2) Non-pseudogenes with fragments per kilobase million (FPKM) value >4; (3) Length <1500-nt. Among the top five candidates with high FPKM value (Supplementary Fig. S1B), SNHG17 is the only one that was upregulated in all ten malignancies analyzed based on the cancer genome atlas (TCGA) data (Fig. 1D; Supplementary Fig. S1C), and a higher SNHG17 level was associated with shorter recurrence-free survival (RFS) and overall survival (OS) of HCC patients (Fig. 1E; Supplementary Fig. S1D). Upregulation of SNHG17 was further confirmed in our HCC cohort (Fig. 1F). Moreover, SNHG17 expression significantly increased during the early and middle G1-phases (Fig. 1A–B). Further analyses characterized that SNHG17 contained 862 nucleotides and six exons (Supplementary Fig. S2A–B), located at both cytoplasm and nucleus (Supplementary Fig. S2C) and had no protein-coding capacity (Supplementary Fig. S2D–E).

c-Myc is a well-known proto-oncogene and early response gene at the G1-phase [3]. Notably, the level of SNHG17 began to increase right after c-Myc elevation, and lowered after c-Myc decline at the G1-phase (Fig. 1B). We therefore examined whether SNHG17 could affect c-Myc level, and found that the protein level of c-Myc was enhanced by overexpressing SNHG17 but was reduced by silencing SNHG17 (Fig. 2A–B). Moreover, silencing SNHG17 accelerated the decline of c-Myc protein during cell cycle progression (Fig. 2C). However, neither knockdown nor overexpression of SNHG17 affected the level of c-Myc mRNA (Supplementary Fig. S3A–B), suggesting that SNHG17 may affect c-Myc level at post-transcriptional level. Subsequent investigations, using cycloheximide to block the de novo protein synthesis, revealed that the half-life of c-Myc protein was significantly decreased by knocking down SNHG17 (Fig. 2D). Furthermore, proteasome inhibitor MG132 abolished the effect of siSNHG17 in reducing c-Myc level (Fig. 2E), and siSNHG17 increased the level of ubiquitylated c-Myc (Fig. 2F), indicating that SNHG17 may increase c-Myc level by preventing ubiquitin-proteasome-dependent degradation of c-Myc protein.

### SNHG17 promotes G1/S transition and cell proliferation by increasing c-Myc level

**Fig. 3:**
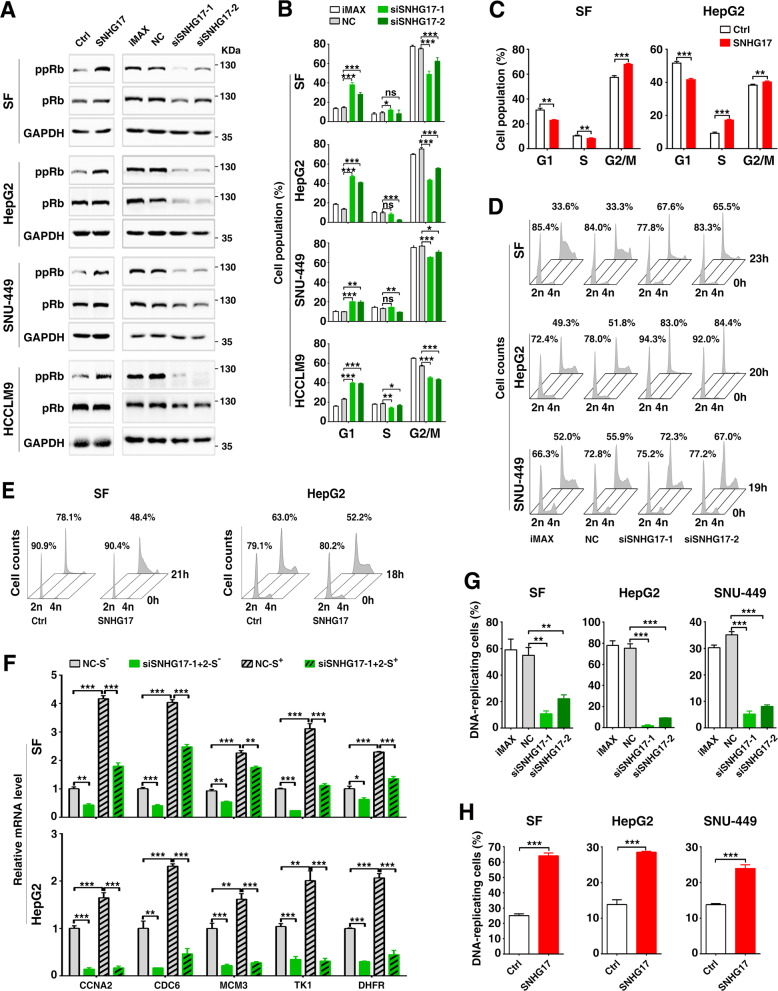
SNHG17 promotes G1/S transition and cell proliferation. **A** The level of phosphorylated-pRb was increased by overexpressing SNHG17 (left panel), but was reduced by silencing SNHG17 (right panel). Cells stably expressing SNHG17 and the control cells (Ctrl) (left panel), or the siRNA-transfected cells (right panel) were subjected to Western blotting. Different exposure times were used to achieve appropriate signal-to-noise for overexpression and knockdown experiments. ppRb, S780-phosphorylated pRb; pRb, total pRb protein. **B**–**C** The fraction of cells at the G1-phase was increased by silencing SNHG17 but was reduced by overexpressing SNHG17. NC- or siSNHG17-transfectants (**B**) and cells stably expressing SNHG17 and the control cells (Ctrl) (**C**) were synchronized with nocodazole before cell cycle analysis. **D**–**E** The serum-stimulated S-phase entry was inhibited by silencing SNHG17 but was promoted by overexpressing SNHG17. NC- or siSNHG17-transfectants (**D**) and cells stably expressing SNHG17 and the control cells (Ctrl) (**E**) were serum-deprived and then grown in the 15% FBS-containing medium for the indicated time periods before cell cycle analysis. The percentage of cells at the G1-phase is indicated for each sample. **F** Silencing SNHG17 inhibited the expression of S-phase genes. NC- or siSNHG17-transfectants were serum-deprived (S^-^) and then cultured in the 15% FBS-containing medium (S^+^) for the indicated time periods before qPCR. **G**–**H** The fraction of DNA-replicating cells was decreased by silencing SNHG17 but was increased by overexpressing SNHG17. Cells undergoing DNA replication were detected using EdU incorporation assays. For **B**–**C** and **F**–**H**, error bars represent mean ± SEM from three independent experiments. *P* values were assessed by unpaired Student’s *t* test. *, *P* < 0.05; **, *P* < 0.01; ***, *P* < 0.001; *ns*, not significant.

Considering that c-Myc plays a critical role in G1/S transition, we first investigated whether SNHG17 could regulate cell cycle progression. The levels of the key regulators of G1/S transition, including cyclin D, cyclin E, cyclin A, CDK4, CDK6, CDK2, p15, p16, p21, pRb, phosphorylated pRb (ppRb), and E2F1 were examined upon SNHG17 overexpression. As shown, the protein levels of ppRb and the c-Myc downstream target genes (CDK4, CDK2, cyclin A2) were significantly increased in SNHG17-overexpressing cells (Fig. 3A, left panel; Supplementary Fig. S4), whereas the level of ppRb was decreased in siSNHG17-transfectants (Fig. 3A, right panel). Consistent with the observed molecular events, nocodazole-synchronized model revealed that the G1-population was significantly increased by silencing SNHG17 (Fig. 3B; Supplementary Fig. S5A) but was reduced by overexpressing SNHG17 (Fig. 3C; Supplementary Fig. S5B). Serum starvation-stimulation assays further showed that the entry of cells into the S-phase was obviously delayed in SNHG17-silencing transfectants (Fig. 3D) but was accelerated in SNHG17-overexpressing cells (Fig. 3E). Moreover, SNHG17 knockdown decreased the mRNA levels of genes essential for S-phase entry (Fig. 3F). Consistently, the fraction of DNA-replicating cells was reduced by siSNHG17 (Fig. 3G; Supplementary Fig. S5C) but was enhanced by overexpressing SNHG17 (Fig. 3H; Supplementary Fig. S5D). These results suggest that SNHG17 may promote G1/S transition and, in turn, cell proliferation.

**Fig. 4:**
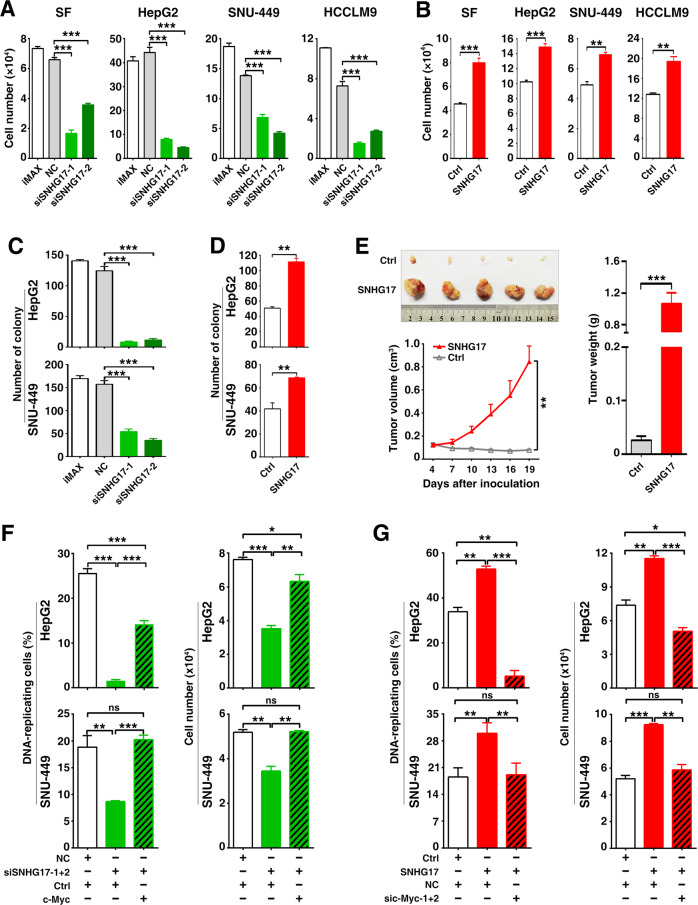
SNHG17 promotes tumor cell growth via c-Myc. **A**–**B** The cell growth was inhibited by silencing SNHG17 but was promoted by overexpressing SNHG17. NC- or siSNHG17-transfectants (**A**) and cells stably expressing SNHG17 and the control cells (Ctrl) (**B**) were subjected to cell counting assays. **C**–**D** The colony formation of hepatoma cells was suppressed by silencing SNHG17 but was promoted by overexpressing SNHG17. **E** The tumor growth was promoted by overexpressing SNHG17 in vivo. HCCLM9 cells stably expressing SNHG17 and the control cells (Ctrl) were subcutaneously injected into NCG mice (*n* = 5). **F** Ectopic expression of c-Myc antagonized the roles of siSNHG17 in decreasing DNA-replicating cells (left panel) and cell growth (right panel). Cells stably expressing c-Myc and the control cells (Ctrl) were co-transfected with the indicated siRNA duplex and then subjected to EdU incorporation assays (left panel) or cell counting (right panel). **G** Silencing c-Myc abrogated the roles of SNHG17 in increasing DNA-replicating cells (left panel) and cell growth (right panel). Cells stably expressing SNHG17 and the control cells (Ctrl) were co-transfected with the indicated siRNA duplex and then subjected to EdU incorporation assays (left panel) or cell counting (right panel). For **A**–**D** and **F**–**G**, error bars represent mean ± SEM from three independent experiments. *P* values were assessed by unpaired Student’s *t* test (**A**–**D**; **E**, right; **F**–**G**), or two-way ANOVA (**E**, left). *, P < 0.05; **, P < 0.01; ***, P < 0.001; *ns*, not significant.

We further evaluated whether SNHG17 regulated tumor growth. As shown, the cell number was significantly decreased by silencing SNHG17 (Fig. 4A) but was increased by overexpressing SNHG17 (Fig. 4B). Consistently, the siSNHG17-transfected hepatoma cells displayed reduced colony formation than the NC-transfectants (Fig. 4C; Supplementary Fig. S6A), while the SNHG17-overexpressing hepatoma cells formed more colonies (Fig. 4D; Supplementary Fig. S6B). Mouse xenograft models revealed that tumors overexpressing SNHG17 grew faster than control xenografts (Fig. 4E), suggesting that SNHG17 may function as an oncogenic lncRNA to promote tumor growth.

Next, we validated whether SNHG17 exerted its function via c-Myc, and found that overexpressing c-Myc attenuated the roles of siSNHG17 in repressing G1/S transition and cell growth (Fig. 4F), whereas inhibiting c-Myc abrogated the stimulatory effects of SNHG17 in G1/S transition and cell growth (Fig. 4G), suggesting that SNHG17 may promote G1/S transition and cell proliferation by stabilizing c-Myc protein.

### SNHG17 enhances the c-Myc level and cell proliferation by directly interacting with LRPPRC

**Fig. 5:**
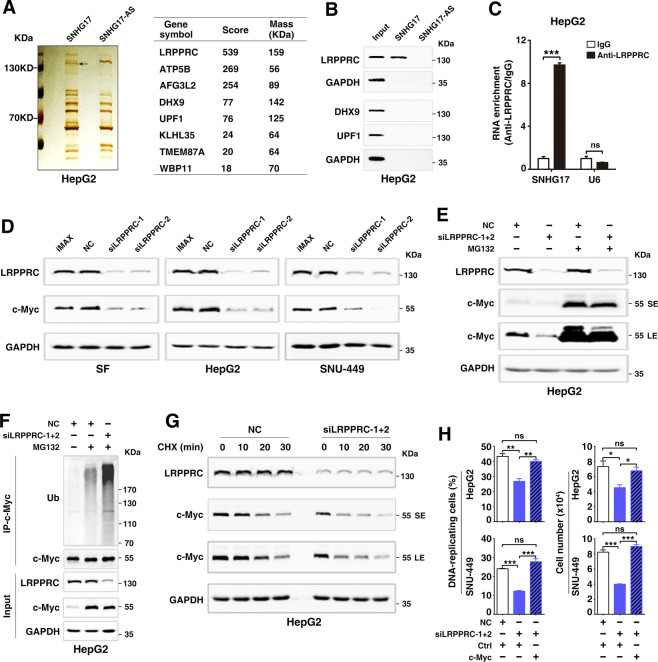
LRPPRC interacts with SNHG17 and promotes G1/S transition by increasing the stability of c-Myc protein. **A** Identification of SNHG17-associated proteins by RNA pulldown and mass spectrometry assays. Biotin-labeled SNHG17 or its antisense RNA (SNHG17-AS, negative control) was incubated with lysates from HepG2 cells to pull down the SNHG17-associated proteins, followed by silver staining. A specific fragment (indicated by an arrow), pulled down by SNHG17 but not SNHG17-AS (left panel), was subjected to mass spectrometry. The candidate proteins are listed (right panel). **B** LRPPRC but not DHX9 or UPF1 was enriched in the protein complexes pulled down by SNHG17. Cellular proteins were pulled down with biotin-labeled SNHG17 or SNHG17-AS and then subjected to Western blotting. GAPDH, negative control. **C** RNA immunoprecipitation assays revealed an interaction between SNHG17 and LRPPRC in vivo. The RNAs that were precipitated by anti-LRPPRC antibody or isotype-matched control IgG were analyzed by qPCR, using primers for SNHG17 or U6. The enrichment was normalized to the IgG control. U6 was used as a negative control. **D** Silencing LRPPRC decreased the protein level of c-Myc. **E** The siLRPPRC-induced c-Myc reduction was abrogated by proteasome inhibitor. NC- or siLRPPRC-transfectants without (−) or with (+) 10 μg/mL MG132 treatment for 3 h were subjected to Western blotting. SE, short exposure; LE, long exposure. **F** Silencing LRPPRC increased the level of ubiquitinated c-Myc. NC- or siLRPPRC-transfectants without (−) or with (+) 10 μg/mL MG132 treatment for 3 h were subjected to immunoprecipitation (IP) using anti-c-Myc antibody and then Western blotting using anti-Ubiquitin antibody (Ub). **G** Silencing LRPPRC decreased the stability of c-Myc protein. NC- or siLRPPRC-transfected cells were treated with 25 μg/mL cycloheximide for the indicated time periods before Western blotting. **H** Ectopic expression of c-Myc antagonized the roles of siLRPPRC in decreasing DNA-replicating cells (left panel) and cell growth (right panel). Cells stably expressing c-Myc and the control cells (Ctrl) were transfected with NC or siLRPPRC and then subjected to EdU incorporation assays (left panel) and cell counting (right panel). For **C** and **H**, error bars represent mean ± SEM from three independent experiments. *P* values were assessed by unpaired Student’s *t* test. *, P < 0.05; **, *P* < 0.01; ***, *P* < 0.001; *ns*, not significant.

To explore how SNHG17 upregulated c-Myc, RNA pulldown assays were used to identify the SNHG17-associated proteins. According to the mass spectrum assay, three candidate proteins, including LRPPRC, DHX9, and UPF1, had high scores and appropriate molecular weight (Fig. 5A). Notably, only LRPPRC, a leucine-rich PPR cassette protein with RNA-binding activity, was pulled down by SNHG17 but not its antisense RNA (Fig. 5B). Moreover, RNA immunoprecipitation assays revealed that compared with the IgG-control group, the LRPPRC-precipitated complex contained more SNHG17 but a similar amount of negative control U6 RNA (Fig. 5C; Supplementary Fig. S7), confirming the interaction between SNHG17 and LRPPRC. However, silencing SNHG17 did not affect the protein level (Supplementary Fig. S8A) and subcellular localization (Supplementary Fig. S8B) of LRPPRC; and silencing LRPPRC had no impact on the level (Supplementary Fig. S8C) and subcellular localization of SNHG17 (Supplementary Fig. S8D). Furthermore, we did not detect an interaction between the protein/mRNA of c-Myc and SNHG17 or LRPPRC protein (Supplementary Fig. S9A–C).

LRPPRC has been shown to regulate the expression of genes at the transcriptional and post-transcriptional levels by binding to its target RNAs [20–24]. It is unknown whether LRPPRC regulates the stability of c-Myc protein or G1/S transition. We found that similar to the effects of siSNHG17, siLRPPRC had no impact on the c-Myc mRNA level (Supplementary Fig. S9D) but observably decreased the level of c-Myc protein (Fig. 5D), and this effect was attenuated by MG132 treatment (Fig. 5E). Furthermore, siLRPPRC increased the polyubiquitination (Fig. 5F) and reduced the stability of c-Myc protein (Fig. 5G), indicating that LRPPRC may enhance c-Myc level via a post-translational regulatory mechanism. Moreover, siLRPPRC inhibited G1/S transition and cell growth, which was abrogated by overexpressing c-Myc (Fig. 5H). These data suggest that LRPPRC may promote G1/S transition and cell proliferation by increasing the stability of c-Myc protein.

**Fig. 6:**
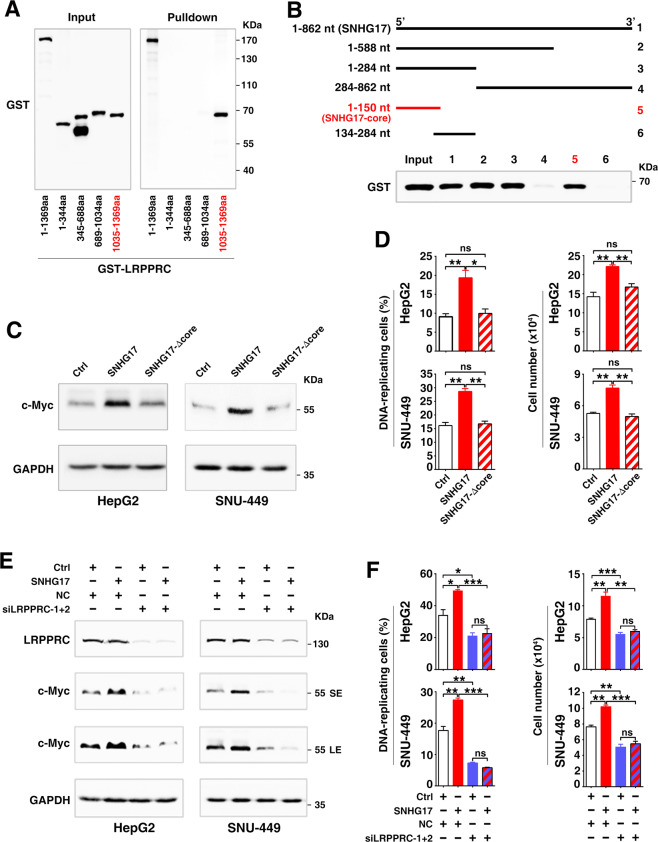
SNHG17 increases c-Myc level and promotes G1/S transition via interacting with LRPPRC. **A** SNHG17 was associated with the 1035–1369-aa domain of LRPPRC. Biotin-labeled SNHG17 was incubated with GST-tagged full-length or truncated LRPPRC, then pulled down by streptavidin-beads, and the retrieved proteins were detected by Western blotting. **B** The 1–150-nt fragment of SNHG17 bound to the 1035–1369-aa domain of LRPPRC. Biotin-labeled full-length or truncated SNHG17 was incubated with the GST-tagged 1035–1369-aa of LRPPRC, then pulled down by streptavidin-beads, and the retrieved proteins were detected by Western blotting. **C**–**D** Deletion in the 1–150-nt of SNHG17 attenuated the roles of SNHG17 in increasing c-Myc protein level, DNA-replicating cells, and cell growth. Cells stably expressing SNHG17 or 1–150-nt-deleted mutant (SNHG17-Δcore) and the control cells (Ctrl) were subjected to Western blotting (**C**), EdU incorporation assays (**D**, left panel), or cell counting (**D**, right panel). **E**–**F** Silencing LRPPRC abrogated the roles of SNHG17 in increasing c-Myc level, DNA-replicating cells, and cell growth. Cells stably expressing SNHG17 and the control cells (Ctrl) were transfected with NC or siLRPPRC, then subjected to Western blotting (**E**), EdU incorporation assays (**F**, left panel) or cell counting (**F**, right panel). SE, short exposure; LE, long exposure. For **D** and **F**, error bars represent mean ± SEM from three independent experiments. *P* values were assessed by unpaired Student’s *t* test. *, *P* < 0.05; **, P < 0.01; ***, *P* < 0.001; *ns*, not significant.

Next, we explored whether SNHG17 increased c-Myc level by binding to LRPPRC. As shown, only the full-length LRPPRC and the fragments containing the 1035–1369-aa could be pulled down by SNHG17 (Fig. 6A), suggesting the 1035–1369-aa as an essential domain for LRPPRC to bind SNHG17. Further study revealed that only full-length SNHG17 and the fragments carrying the 1–150-nt region could pull down the GST-LRPPRC-1035-1369-aa fragment (Fig. 6B), indicating 1–150-nt as the core sequence (SNHG17-core) for SNHG17 to bind the 1035–1369-aa domain of LRPPRC. Notably, the roles of SNHG17 in increasing c-Myc level and promoting G1/S transition and cell growth were attenuated by deleting the 1–150-nt sequence of SNHG17 (Fig. 6C, D) or by silencing LRPPRC (Fig. 6E, F; Supplementary Fig. S10A, B). These results suggest that SNHG17 may exert its function via LRPPRC.

**Fig. 7:**
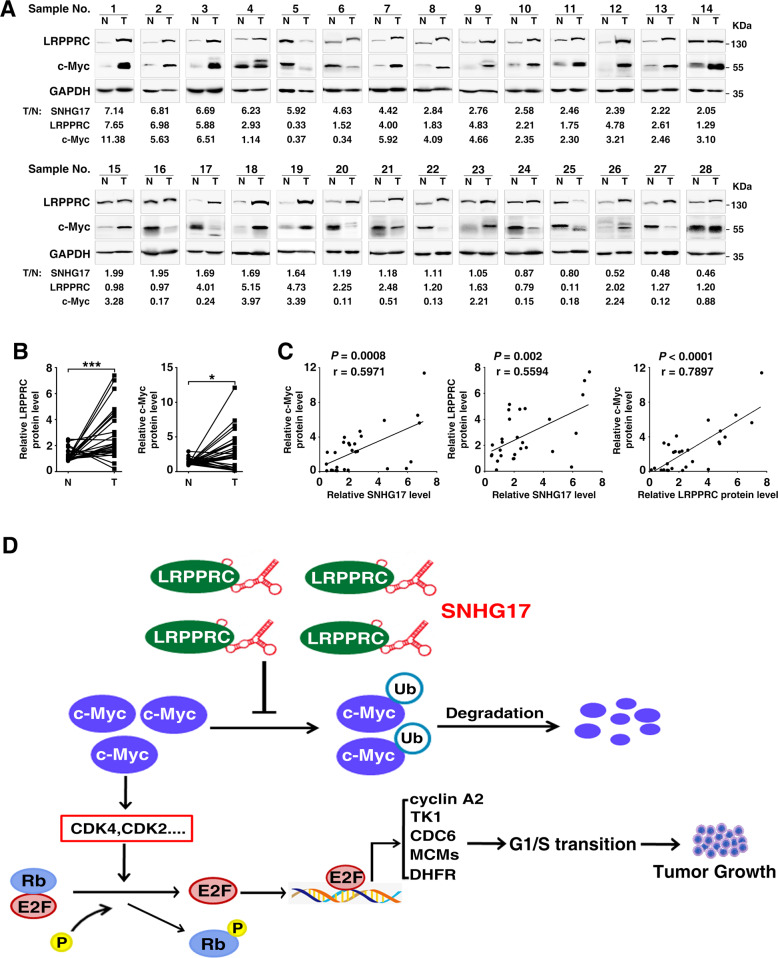
The level of SNHG17 is positively correlated with that of LRPPRC and c-Myc in human HCC tissues. **A** Expression of SNHG17, LRPPRC, and c-Myc protein in paired HCC (T) and adjacent non-tumor livers (N). The protein level of LRPPRC or c-Myc was assessed by Western blotting. The intensity of each band was quantified. The band intensity of LRPPRC and c-Myc in each sample was normalized to that of GAPDH (internal control). The values under each pair of samples indicate the fold change of the normalized levels of the indicated molecules in HCC, relative to that in the matched non-tumor livers. The normalized RNA levels of SNHG17 were derived from Fig. 1F. **B** The protein levels of LRPPRC and c-Myc increased in HCC tissues. Quantified protein levels in paired HCC (T) and adjacent non-tumor livers (N) from Fig. 7A were compared. **C** Positive correlation among the expression levels of SNHG17, LRPPRC, and c-Myc in human HCC tissues. **D** Working model of the SNHG17/LRPPRC/c-Myc regulatory axis. *P* values were assessed by paired Student’s *t* test (**B**), or Pearson’s correlation coefficient (**C**). *, *P* < 0.05; ***, *P* < 0.001.

We then validated the in vitro findings in human samples. As shown, the levels of SNHG17, LRPPRC, and c-Myc were significantly increased in HCC tissues (Fig. 7A, B), and they showed a positive correlation with each other (Fig. 7C). Similar to the results of SNHG17 (Fig. 1D, E), LRPPRC was upregulated in different types of cancer and high LRPPRC level was associated with worse survival of HCC patients (Supplementary Fig. S11A, B).

Taken together, SNHG17 may inhibit c-Myc ubiquitination and thus stabilize c-Myc protein by associating with LRPPRC, which leads to upregulation of c-Myc, acceleration of G1/S transition and cell proliferation, and consequent tumor growth (Fig. 7D).

## Discussion

Aberrant overexpression of c-Myc oncogene, which is frequently reported in a variety of tumors, contributes to the dysregulation of G1/S transition and is a key event in tumor development [3, 25, 26]. The roles of lncRNAs in c-Myc regulation remain largely unknown. In this study, we identify an oncogenic lncRNA SNHG17 that is upregulated at the early G1-phase and in various cancer types, including HCC. Moreover, SNHG17 enhances the stability of c-Myc protein by directly binding to LRPPRC and, in turn, promotes G1/S transition and tumor growth.

c-Myc is an early response gene that can be activated by growth signals [27]. In normal cells, c-Myc level is tightly regulated at transcriptional and post-transcriptional levels. c-Myc protein has a short half-life of about 30 min. It undergoes ubiquitin proteasome-mediated degradation soon after it is activated [25, 28–30]. Post-translational modifications, including phosphorylation, sumoylation, and acetylation [31–33] are well-known mechanisms that regulate c-Myc degradation and ensure the proper termination of growth signals. It is reported that aberrations in c-Myc regulators, like CAMKIIγ [31], SENP1 [34], and HDAC3 [33], result in accumulation of c-Myc and overexpression of c-Myc target genes, leading to unlimited proliferation and malignant transformation. Here, we showed that SNHG17 enhanced the stability of c-Myc protein based on the following evidence. Firstly, c-Myc protein level was increased by overexpressing SNHG17 but was reduced by silencing SNHG17. However, neither knockdown nor overexpression of SNHG17 affected the level of c-Myc mRNA. Secondly, silencing SNHG17 increased the ubiquitylated c-Myc level and accelerated the decline of c-Myc protein, whereas proteasome inhibitor MG132 abolished the effect of siSNHG17 in decreasing c-Myc protein level. Thirdly, SNHG17 and c-Myc exhibited a similar expression pattern during cell cycle progression, and upregulation of SNHG17 was significantly correlated with elevation of c-Myc protein in HCC tissues. Our findings suggest that lncRNAs represent a new class of c-Myc regulators, and upregulation of SNHG17 in tumors may facilitate tumor growth via c-Myc.

As a snoRNA host gene-derived lncRNA, SNHG17 was upregulated in various cancer types, and its upregulation predicts a poor survival. Genomic amplification was reported as one cause of SNHG17 overexpression in non-small-cell lung cancer (NSCLC), and knockdown of SNHG17 inhibited the proliferation and migration and promoted the apoptosis of NSCLC cells with unknown mechanisms [35]. It was shown that helicobacter pylori infection elevated SNHG17 expression in gastric cancers, resulting in increased genome instability via a SNHG17/miR-3909/RING1/Rad51 pathway [36]. Zhang et al. showed that SNHG17 promoted cell proliferation, migration, invasion, and resistance to apoptosis by polycomb repressive complex 2-mediated epigenetic repression on cyclin-dependent kinase inhibitors, including p15 and p57 [37]. Although SNHG17 has been implicated in tumor development, its role in regulating G1/S transition via c-Myc has not been reported yet. We found that SNHG17 promoted G1/S transition and, in turn, cell proliferation and tumor growth by increasing the level of c-Myc protein. SNHG17 stabilized c-Myc protein via binding to the 1035–1369-aa domain of LRPPRC. We further characterized the 1–150-nt fragment as the essential domain for the function of SNHG17 because deletion of this region abrogated the interaction between SNHG17 and LRPPRC, and attenuated the effects of SNHG17 in enhancing c-Myc stability, G1/S transition, and cell growth.

LRPPRC is an RNA binding protein that regulates gene translation, RNA polyadenylation and transport, and maintains RNA and genome stability [20–24]. Recently, Chen and colleagues reported that lncRNA DANCR directly interacted with LRPPRC and guided LRPPRC protein to bind and stabilize the mRNAs of CCND1, PLAU, and IL-11, resulting in enhanced proliferation, migration, and invasion of bladder cancer cells [24]. These findings suggest that different lncRNAs may interact with LRPPRC protein to increase the mRNA or protein stability of different genes, which highlight the importance of LRPPRC in the regulation of gene expression.

The role of LRPPRC in tumor development remains inconclusive. It has been reported that LRPPRC protein levels were significantly reduced in diethylnitrosamine (DEN)-induced mouse HCC tissues, and liver-specific deletion of LRPPRC increased incidence of DEN-induced HCC [23]. However, LRPPRC is upregulated in human lung adenocarcinoma [38], and knockdown of LRPPRC inhibits the growth of bladder and lung cancer cells in vitro and in vivo [24, 38]. Consistently, we found significant upregulation of LRPPRC in human HCC samples. Analysis using the cancer genome atlas (TCGA) data showed that the mRNA levels of LRPPRC were significantly increased in 8 out of 10 malignancies, including HCC. And high LRPPRC expression was correlated with worse survival of HCC patients. It has been shown that the gene mutation profiles are quite different between DEN-induced HCC and human HCC [39]. The discrepancy between studies in human and mice might be attributed to the species specificity and the different pathological processes in human tumors and chemical-induced tumors in mice.

It has been reported that LRPPRC can suppress autophagy [40, 41] and c-Myc protein can be degraded via autophagy pathway [42], which raises the possibility that LRPPRC may regulate the autophagic degradation of c-Myc protein. However, we found that autophagy inhibitor chloroquine could not abolish the effect of siLRPPRC in decreasing c-Myc protein level (Supplementary Fig. S12). On the other hand, silencing LRPPRC increased the ubiquitylated c-Myc level and reduced the stability of c-Myc protein, suggesting that LRPPRC may regulate c-Myc stabilization through ubiquitin-proteasome system. However, we did not detect an association between LRPPRC and the mRNA or protein of c-Myc, suggesting that LRPPRC may act in an indirect manner, for example, through some E3 ligases to control the ubiquitination of c-Myc. Our study discloses the novel functions of LRPPRC and explores new mechanisms responsible for the tumor-promoting effect of LRPPRC.

In conclusion, we identify a SNHG17/LRPPRC/c-Myc regulatory axis and characterize its function in G1/S transition, which uncovers new mechanisms underlying cell proliferation and tumor growth, and provides potential targets for anti-cancer therapy.

## Materials and methods

Additional information is provided in the Supplementary Materials and Methods.

### Human tissues

Human HCC tissues and adjacent noncancerous liver tissues were collected from patients who undertook tumor resection at the Cancer Center, Sun Yat-sen University, P.R. China. All tissues were histologically examined. No local or systemic therapy had been conducted before surgery. After surgical resection, no other anti-cancer therapy was carried out before recurrence. Informed consent was obtained from each patient, and the protocol was approved by the Institutional Research Ethics Committee.

### Cell lines and cell culture

Transformed human embryonic kidney cell line, HEK293T (ATCC, CRL-3216), and three human hepatoma cell lines, HepG2 (ATCC, HB-8065), SNU-449 (ATCC, CRL-2234), and HCCLM9 [43, 44] were cultured in Dulbecco’s modified Eagle’s medium (DMEM) (Corning, Manassas, VA, USA) containing 10% fetal bovine serum (FBS) (Gibco, Thermo Fisher Scientific, Waltham, Massachusetts, USA), 100 mg/mL streptomycin and 100 U/mL penicillin in a humidified atmosphere with 5% CO_2_ at 37 °C. Human primary SF cells were isolated from human neonatal foreskin as described previously [45] and cultured in RPMI 1604 medium (Corning) supplemented with 10% FBS (Gibco), 100 mg/mL streptomycin, and 100 U/mL penicillin in a humidified atmosphere with 5% CO_2_ at 37 °C. The cell lines with stable expression of SNHG17 (SF-SNHG17, HepG2-SNHG17, SNU-449-SNHG17, HCCLM9-SNHG17), SNHG17-Δcore (HepG2-SNHG17-Δcore, SNU-449-SNHG17-Δcore), c-Myc (HepG2-c-Myc, SNU-449-c-Myc), and their matched control lines (SF-Ctrl, HepG2-Ctrl, SNU-449-Ctrl, HCCLM9-Ctrl) were established by infecting cells with lentivirus that expressed the target sequence, followed by selection under puromycin (1 μg/mL) for 6 days before used. All these sublines expressed copGFP.

### RNA oligoribonucleotides

The small interfering RNAs (siRNAs) targeting the transcripts of human SNHG17 (GeneBank accession No. NR_152762.1), c-Myc (NM_002467.6) and LRPPRC (NM_133259.4) were designated as siSNHG17, sic-Myc, and siLRPPRC, respectively, and were purchased from Ribobio (Guangzhou, China). The negative control (NC) RNA duplex for siRNA was non-homologous to any human genome sequences.

### Rapid amplification of cDNA ends (RACE)

Total RNA of SF cells was used to amplify 5′- and 3′-ends of SNHG17. The 5′-end was determined using a 5′-Full RACE Kit (D315, TaKaRa, Kyoto, Japan). 3′RACE was performed as described previously [45]. The PCR-amplified sequences of 5′- and 3′-end fragments were determined by direct sequencing.

### Vector construction

The following expression vectors were used: the lentivirus expression vectors, including pCDH-SNHG17, pCDH-SNHG17-Δcore, pCDH-SNHG17-ORF1-flag, pCDH-SNHG17-ORF2-flag, pCDH-SNHG17-ORF3-flag, pCDH-c-Myc, pCDH-MPM-flag [46]; the pcDNA3.0-puro expression vectors, including pc3-SNHG17, pc3-SNHG17-antisense, pc3-SNHG17/1-588 bp, pc3-SNHG17/1-284 bp, pc3-SNHG17/1-150 bp, pc3-SNHG17/134-284 bp, pc3-SNHG17/284-862 bp; the prokaryotic vectors expressing GST fusion proteins, including pGEX-LRPPRC, pGEX-LRPPRC/1-344aa, pGEX-LRPPRC/345-688aa, pGEX-LRPPRC/689-1034aa, pGEX-LRPPRC/1035-1369aa.

### RNA sequencing and analysis

Total RNA was isolated from SF cells using Trizol reagent (Invitrogen). The quality of extracted RNA was assessed using the NanoDrop ND-2000 spectrophotometer. Directional RNA-seq libraries were constructed and then sequenced at the Novogene Bioinformatics Institute (Beijing, China) on an Illumina HiSeq-PE150 Platform. The obtained reads were then mapped to the human genome (hg38) database using STAR v.2.6.0, and fragments per kilobase of exon per million mapped reads (FPKM) were calculated using RSEM. Gene expression levels and differential expression were quantified with Cuffdiff. The RNA-seq data have been deposited in the Gene Expression Omnibus (GEO) database under accession number GSE166448.

### Analysis of gene expression

The RNA and protein levels of gene were analyzed by real-time quantitative polymerase chain reaction (qPCR) and Western blotting, respectively.

The sequences of all oligonucleotides used for siRNA, qPCR, RACE, and vector construction are listed in Table S1.

### Cell transfection and lentivirus production

Reverse transfection of RNA oligoribonucleotides was performed with Lipofectamine RNAiMAX (Invitrogen). A final concentration of 10 nM duplex was used. Plasmid DNAs were transfected using Lipofectamine 3000 (Invitrogen).

To produce lentivirus, HEK293T cells were co-transfected with lentivirus expression vector containing target sequence and packing plasmids (pMD2.G and psPAX2, Addgene MA, USA) by calcium phosphate precipitation, then refreshed with culture medium 16 h after transfection. Thirty-six hours later, the lentivirus supernatant was harvested and stored in aliquots at −80 °C until use. Target cells were infected with lentiviral supernatant supplemented with 8 μg/mL polybrene (Millipore, Billerica, MA).

### Cell cycle analysis

All cell cycle analyses were performed using a detergent-containing hypotonic solution (Krishan’s reagent: 0.3% NP-40, 0.1% sodium citrate, 0.05% sodium chloride, 0.05 mg/mL propidium iodide, 0.02 mg/mL ribonuclease A) and fluorescence activated cell sorting (FACS) (Gallios, Beckman Coulter, Miami, FL, USA) as previously described [45].

For the nocodazole-synchronized model, cells were treated with 50 ng/mL nocodazole before FACS analysis. For the serum starvation-stimulation model, cells were serum-deprived for 48 h, followed by serum re-addition, and then harvested at the indicated time points.

### The 5-ethynyl-2′-deoxyuridine (EdU) incorporation assay

EdU incorporation assay was conducted using Cell-Light EdU Apollo^®^ 567 In Vitro Kit (C10310-1, Ribobio). Briefly, 12 h after last transfection or seeding, cells were serum-deprived for 48 h and then cultured in 15% FBS-containing medium for the indicated time periods, followed by incubation in complete medium containing 50 μM EdU for additional 2 h. Cells were then fixed with 4% paraformaldehyde, and sequentially incubated with 2 mg/mL glycine for 5 min, 1 × PBS containing 0.5% Triton X-100 for 10 min, and click reaction buffer (containing Apollo^®^ 567) for 30 min. Nuclei were counterstained with Hoechst 33342. The fraction of DNA-replicating cells, which represented for cell proliferation rate, was expressed as the ratio of EdU-positive cells to the number of Hoechst 33342-positive cells. At least 500 cells were counted for each sample.

### Cell counting assay

Cell counting assay was applied to evaluate cell growth. For loss-of-function assay, SF (1 × 10^4^), HepG2 (4.5 × 10^4^), SNU-449 (1 × 10^4^), and HCCLM9 (2 × 10^4^) cells transfected with the indicated siRNAs were grown in a 24-well plate for 4 days before cell counting by Countstar (ALIT Life Sciences, Shanghai, China). For gain-of-function assay, SF (5 × 10^3^), HepG2 (1.5 × 10^4^), SNU-449 (5 × 10^3^), and HCCLM9 (2 × 10^4^) sublines stably expressing the indicated genes were seeded in a 24-well plate for 4 days before cell counting by Countstar (ALIT Life Sciences).

### Colony formation assay

For the loss-of-function assay, NC- or siSNHG17-transfected cells (1000 cells for HepG2 and 300 cells for SNU-449) were placed in a 6-well plate and maintained in complete medium for 14 (HepG2) or 10 (SNU-449) days. For the gain-of-function assay, HepG2 (500 cells) and SNU-449 (150 cells) sublines stably expressing SNHG17 and their control lines were seeded in a 6-well plate for 14 (HepG2) or 12 (SNU-449) days. The colonies were fixed with methanol and then stained with 0.1% crystal violet in 20% methanol for 15 min before analysis.

### Mouse xenograft models

Male NOD-Prkdcem26Cd52Il2rgem26Cd22/Nju (NCG) mice at 4 weeks of age were used. HCCLM9-SNHG17 and its control line HCCLM9-Ctrl (6.0 × 10^6^) resuspended in 100 μL of DMEM/matrigel (1:1 volume; R&D Systems, Minneapolis, MN, USA) were subcutaneously injected into the right and left side of the posterior flank, respectively. Mice were sacrificed 20 days after implantation. Tumor growth was measured every three days, and the volume of the tumor was detected with electronic digital calipers and calculated with the formula: volume = (length × width^2^)/2. At the end of experiments, tumors were dissected, photographed, and weighed.

All mouse experiments were approved by the Institutional Animal Care and Use Committee at Sun Yat-sen University. All experiments were performed according to the Guide for the Care and Use of Laboratory Animals (NIH publications Nos. 80-23, revised 1996) and following the institutional ethical guidelines for animal experiments.

### Isolation of subcellular fraction

The cytoplasmic and nuclear extracts were isolated using NE-PER Nuclear and Cytoplasmic Extraction Reagents (Pierce, Rockford, IL, USA), followed by isolation of RNAs or proteins.

### Purification of GST-fusion proteins

GST-tagged full-length or truncated mutants of human LRPPRC was expressed in *Escherichia coli* (*E. coli*) BL21 (DE3, TaKaRa). GST-fusion proteins were induced with IPTG and purified by GST-sefinose (TM) resin (P2251, Beyotime, Shanghai, China), then concentrated in BC100 buffer (20 mM Tris-HCl at pH 8.0, 0.5 mM EDTA at pH 8.0, 100 mM KCl, 20% glycerol, 0.5 mM DTT and 1 × protease inhibitor cocktail) by Amicon Ultra-15 Centrifugal Filter Devices (UFC901008, Millipore) and stored at −80 °C until use. The protein concentration was measured by BCA Protein Assay Kit (P0012S, Beyotime).

### RNA pulldown assay

RNA pulldown assay was performed as previously reported [45]. In brief, full-length or truncated mutants of human SNHG17 or antisense of SNHG17 were in vitro transcribed and biotin-labeled using Biotin RNA labeling mix (2147483647, Roche, Mannheim, Germany), then incubated with purified GST-fusion proteins or with HepG2 cell lysates that were pre-cleared by yeast tRNA at RT for 1 h. After incubation with pre-washed streptavidin MagneSphere^®^ paramagnetic particles (Promega, Madison, USA) for 30 min at RT, the biotin-labeled RNA–protein complexes with paramagnetic particles were collected by a magnetic holder and washed with IP-lysis buffer (25 mM Tris-HCl at pH 7.4, 150 mM NaCl, 1 mM EDTA at pH 8.0, 1% NP-40, 5% glycerol and 100 U/mL RNase inhibitor) for 5 times, then digested with RNase A (Thermo Scientific, Waltham, MA, USA) in 10 mM Tris-HCl (pH 8.0) at 37 °C for 15 min to release the RNA binding proteins. The retrieved proteins were separated by SDS-PAGE gel, followed by silver staining, and analyzed by mass spectrometry (Beijing Protein Innovation, Beijing, China) or Western blotting.

### Immunoprecipitation (IP) assay

IP assay was carried out as previously [47] reported with modifications. Briefly, HepG2 cell lysates were pre-cleared with yeast tRNA and then incubated with the indicated antibody or isotype-matched IgG at 4 °C for 4 h. Then the immunoprecipitated complexes were collected by incubation with protein A/G magnetic beads (B23201; Bimake, Houston, TX, USA) at 4 °C for 2 h. After washing with IP lysis buffer for 5 times, the immunoprecipitated complexes were eluted with 1 × SDS lysis buffer and then analyzed by Western blotting.

### RNA immunoprecipitation assay

The LRPPRC-RNA complex was immunoprecipitated by anti-LRPPRC antibody or isotype-matched IgG (negative control). RNA was extracted from the precipitates by TRIzol reagent (Invitrogen) and detected by qPCR.

### Bioinformatics

The potential coding capacity of SNHG17 transcript was predicted using ATG^pr^ (http://atgpr.dbcls.jp/). Kaplan–Meier survival analysis for HCC patients with high or low gene expression level was performed through GEPIA website (http://gepia.cancer-pku.cn/detail.php?gene). The expression data of SNHG17 and LRPPRC in various tumors were obtained from the TCGA database and downloaded from the TANRIC websites (https://ibl.mdanderson.org/tanric/_design/basic/main.html) and the UCSC Xena database (https://xena.ucsc.edu/public/), respectively.

### Statistical analysis

Paired Student’s *t* test was employed to analyze the difference in gene expression levels between paired HCC tissues and adjacent non-tumor liver tissues. Pearson’s correlation coefficient was employed to analyze the correlation among the levels of SNHG17, LRPPRC, and c-Myc in paired HCC tissues and adjacent non-tumor liver tissues.

Data were expressed as the mean ± standard error of the mean (SEM) from at least three independent experiments. The differences between two groups were analyzed using two-tailed unpaired Student’s *t* test or two-way ANOVA. A *P*-value of less than 0.05 was considered statistically significant, and all statistical tests were two-sided. All analyses were performed using GraphPad Prism version 5.0 software (GraphPad Software, Inc., San Diego, CA, USA).

## Supplementary information

Supplementary_information

Supplementary Figure S1

Supplementary Figure S2

Supplementary Figure S3

Supplementary Figure S4

Supplementary Figure S5

Supplementary Figure S6

Supplementary Figure S7

Supplementary Figure S8

Supplementary Figure S9

Supplementary Figure S10

Supplementary Figure S11

Supplementary Figure S12

## Data Availability

The data that support this study are present in the manuscript and supplementary information, and are available from the corresponding author upon request. The RNA-seq data have been deposited in the Gene Expression Omnibus (GEO) database under accession number GSE166448.
