# Supplementary material for: LncRNA SNHG17 interacts with LRPPRC to stabilize c-Myc protein and promote G1/S transition and cell proliferation
Source: Cell Death Dis. 2021 Oct 20;12(11):970. doi: 10.1038/s41419-021-04238-x (PMC8528917; doi:10.1038/s41419-021-04238-x)
Supplement: Supplementary file 1 — Supplementary_information [file 41419_2021_4238_MOESM1_ESM.doc]

**Supplementary information**

**LncRNA SNHG17 Interacts with LRPPRC to Stabilize c-Myc Protein and Promote G1/S Transition and Cell Proliferation**

*Jin-Yu Liu, Ya-Jing Chen, Huan-Hui Feng, Zhan-Li Chen, Yun-Long Wang, Jin-E Yang^*^ and Shi-Mei Zhuang^*^*

**Inventory of supplemental information**1. Supplemental Materials and Methods...………….……..Page 2 - 5
2. Supplemental Figure Legends……….…….……………Page 7 - 10
3. Table S1…………...………………….……...…...…......Page 11-14

**Supplementary Materials and Methods**

**Vector construction**

pCDH-SNHG17 or pCDH-SNHG17(Δcore) was created by respectively inserting the full-length SNHG17 and mutant SNHG17 with 1-150-nt deletion into the *Xba*I/*EcoR*I sites of pCDH-CMV-MCS-EF1-CopGFP-T2A-Puro vector (System Biosciences, Palo Alto, CA, USA), which contains a copGFP expression cassette and is designated as pCDH-Ctrl in this study. To produce pCDH-c-Myc, the coding sequence of c-Myc was inserted into the *Eco*RI/*Bam*HI sites of pCDH-Ctrl.

To determine the protein-coding potential of SNHG17, the predicted open reading frame (ORF) together with its 5’UTR was fused in frame to the N-terminus of flag-tag (without ATG) and cloned into the *Xba*I/*Eco*RI sites of pCDH-Ctrl, and designated as pCDH-flag-SNHG17-ORF1, pCDH-flag-SNHG17-ORF2 or pCDH-flag-SNHG17-ORF3. A plasmid expressing MPM-flag fusion protein was used as a positive control.

To obtain biotin-labeled full-length or truncated mutants of SNHG17, vectors including pc3-SNHG17, pc3-antisense-SNHG17, pc3-SNHG17/1-588bp, pc3-SNHG17/1-284bp, pc3-SNHG17/Δcore, pc3-SNHG17/134-284bp and pc3-SNHG17/284-862bp were generated by respectively cloning the full-length SNHG17, antisense of full-length SNHG17, and the indicated fragment of SNHG17 into the *Eco*RI/*Xba*I sites of pc3-puro and then used as templates for *in vitro* transcription. The backbone plasmid pc3-puro was produced based on the pcDNA3.0 vector (Invitrogen, Carlsbad, CA, USA) in which the neomycin open reading frame was replaced with an expression cassette for the puromycin-resistance gene.

To express GST-LRPPRC fusion protein, the coding sequence of LRPPRC was inserted into the *Bam*HI/*Xho*I sites of the prokaryotic expression vector pGEX-6P-1 (GE Healthcare Bio-Sciences, Pittsburgh, PA, USA), and designated as pGEX-LRPPRC. The plasmids including pGEX-LRPPRC/1-344aa, pGEX-LRPPRC/345-688aa, pGEX-LRPPRC/689-1034aa, and pGEX-LRPPRC/1035-1369aa were generated by respectively cloning the indicating fragment of LRPPRC into the *Bam*HI/*Not*I sites of pGEX-6P-1.

All constructs were verified by sequencing. All sequences of oligonucleotides and primers are listed in Table S1.

**Analysis of gene expression**

The RNA levels were examined by real-time quantitative polymerase chain reaction (qPCR). Total RNA was extracted using TRIzol reagent (Life Technologies, Carlsbad, CA, USA), then reversely transcribed with random primers (B0043, Sangon Biotech, Shanghai, China) using M-MLV Reverse Transcriptase (M1701, Promega), followed by qPCR using 2 × SYBR Green qPCR Master Mix (B21202, Bimake, Houston, TX, USA). All reactions were run in duplicate. The cycle threshold (Ct) values differed by less than 0.5 among the duplicates. The relative level of target gene was normalized to that of internal control gene, which yielded a 2^-ΔCt^ value. U6 and GAPDH were used as the internal control genes for the relative expression levels in tissues and cell lines, respectively.

The protein levels were detected by Western blotting. In brief, protein lysates were separated on a SDS-polyacrylamide gel，transferred to polyvinylidene difluoride (PVDF) membranes (162-0177, Bio-Rad, Hercules, CA, USA), and then sequentially incubated with primary and secondary antibodies. Immunoreactive signals were detected with ECL kit (Thermo scientific, Waltham, MA, USA). The antibodies used included: rabbit monoclonal antibodies (mAb) against cyclin D1 (ab134175, Abcam, Cambridge, MA, USA), cyclin D3 (cat.2936, CST, Beverly, MA, USA), cyclin A2 (ab181591, Abcam), CDK2 (cat.2546, CST), CDK4 (ab199728, Abcam), p16 (ab51243, Abcam), p21 (cat.2947, CST), and ubiquitin (ab134953, Abcam); rabbit polyclonal antibodies against phospho-Ser780 of pRb (cat. 9307, CST), c-Myc (cat.9402, CST), lamin A/C (D120927, BBI, Shanghai, China), cyclin E1 (ENT1176, Elabscience, Wuhan, China), cyclin E2 (cat. 4132, CST), E2F1 (cat. 3742, CST), p15 (ab53034, Abcam), LRPPRC (A3365, ABclonal Biotechnology, Wuhan, China), UPF1 (A1521, ABclonal), and GST (RM1005, Beijing Ray Antibody Biotech, Beijing, China); mouse mAb against pRb (cat. 9309, CST), CDK6 (cat. 3136, CST), and Flag (F1804, Sigma-Aldrich, Louis, MO, USA); mouse polyclonal antibody against GAPDH (BM1623, Boster, Wuhan, China).

**RNA immunoprecipitation (RIP) assay**

HepG2 cells (~2 × 10^7^) were washed with 6 mL of ice-cold 1 × PBS for 3 times, then incubated with 6 mL of 1 × PBS containing 0.5% formaldehyde at RT for 10 minutes to crosslink cellular components, and subsequently incubated with 0.25 M glycine at RT for another 5 minutes to quench the reaction. After washing with 10 mL of ice-cold 1 × PBS for 3 times, the pellets were resuspended in 1 mL of IP-lysis buffer (25 mM Tris-HCl at pH 7.4, 150 mM NaCl, 1 mM EDTA at pH 8.0, 1% NP-40, 5% glycerol and 100 U/mL RNase inhibitor) and incubated with 50 U DNase I at 37 °C for 10 minutes, followed by sonication with a Bioruptor (Diagenode, Liege, Belgium). The supernatant was collected by centrifugation at 16,000 g, 4 °C for 10 minutes, and incubated with 4 μg of anti-LRPPRC antibody or isotype-matched control IgG at 4 °C with rotation for 4 hours, followed by incubation with 35 μl of protein A/G magnetic beads (B23201; Bimake) at 4 °C for another 2 hours to precipitate protein-RNA complexes. The beads were collected by a magnetic holder and washed with 500 μl of high-salt IP-lysis buffer (25 mM Tris-HCl at pH 7.4, 500 mM NaCl, 1 mM EDTA at pH 8.0, 1% NP-40, 5% glycerol and 100 U/mL RNase inhibitor) for 5 times, then resuspended in 50 μl of IP-lysis buffer supplemented with 50 μg of proteinase K, followed by incubation at 55 °C for 1 hour and then at 70 °C for another 45 minutes to reverse protein-RNA crosslink. The RNA was extracted by TRIzol reagent (Invitrogen) and analyzed by qPCR.

**Supplementary Figure legends**

**Supplementary Fig. S1. Screening for oncogenic lncRNAs that may regulate G1/S transition.** (A) The screening workflow for candidate lncRNAs. (B) The FPKM value of candidate lncRNAs. (C) Pan-cancer analysis of SNHG8, SNHG1, MIR503HG, SNHG15 expression based on TCGA data. The relative expression levels of the indicated genes in paired tumor (T) and non-tumor (N) tissues from 10 different cancer types are presented. LIHC, liver hepatocellular carcinoma; BLCA, bladder urothelial carcinoma; BRCA, breast invasive carcinoma; HNSC, head and neck squamous cell carcinoma; KIRC, kidney renal clear cell carcinoma; KIRP, kidney renal papillary cell carcinoma; LUAD, lung adenocarcinoma; LUSC, lung squamous cell carcinoma; PRAD, prostate adenocarcinoma; STAD, stomach adenocarcinoma. (D) Kaplan-Meier survival analysis. The median level of the indicated lncRNAs in all 364 HCC tissues derived from TCGA was chosen as the cut-off value to separate the lncRNA high-level group (n = 182) from the low-level group (n = 182). *P* values were assessed by paired Student’s *t*-test (C), or log-rank test (D). *, *P* < 0.05; **, *P* < 0.01; ***, *P* < 0.001; ns, not significant.

**Supplementary Fig. S2. Characterization of the SNHG17 transcript.** (A) The PCR products of RACE assays. The products (indicated by red arrows) of 5’RACE (*up panel*) and 3’RACE (*down panel*) were obtained by PCR and then sequenced. The nucleotide of the 5’- or 3’-end of SNHG17 transcript is marked by black arrows. (B) The sequence of the full-length SNHG17 transcript. The SNHG17-transcript consists of 6 exons, which are indicated by different colors. (C) SNHG17 was localized in both cytoplasm and nucleus. The cytoplasm and nuclei of cells were fractionated and then subjected to qPCR analysis. (D) The potential open reading frames (ORFs) of SNHG17 were predicted using the ATG^pr^ program (http://atgpr.dbcls.jp/). (E) The SNHG17 transcript had no protein-coding capacity. HepG2 cells were transfected with the indicated plasmids for 48 hours, followed by Western blotting to detect the fusion protein using anti-Flag antibody. MPM is a mitochondria-localized micropeptide and the plasmid expressing MPM-flag was used as a positive control, and the pCDH-Ctrl-Flag (Ctrl) plasmid as a negative control.

**Supplementary Fig. S3. SNHG17 has no impact on the mRNA level of c-Myc.** (A-B) Silencing or Overexpressing SNHG17 had no impact on the mRNA level of c-Myc. NC- or siSNHG17-tranfectants (A), or cells stably expressing SNHG17 and the control cells (Ctrl) (B) were subjected to qPCR analysis. Error bars represent mean ± SEM from three independent experiments. *P* values were assessed by unpaired Student’s *t*-test. *, *P* < 0.05; ns, not significant.

**Supplementary Fig. S4. The impact of SNHG17 overexpression on the protein levels of key regulators of G1/S transition.** HepG2 cells stably expressing SNHG17 and the control cells (Ctrl) were subjected to Western blotting.

**Supplementary Fig. S5. SNHG17 promotes G1/S transition and cell proliferation.** (A-B) The fraction of cells at the G1-phase was increased by silencing SNHG17 but was reduced by overexpressing SNHG17. Representative images for Figure 3B (A) and 3C (B). The percentage of cells at the G1-phase is indicated for each sample. (C-D) The fraction of DNA-replicating cells was decreased by silencing SNHG17 but was increased by overexpressing SNHG17. Representative images for Figure 3G (C) and 3H (D). Scale bar, 25 μm.

**Supplementary Fig. S6. SNHG17 promotes the colony formation of tumor cells.** (A-B) The colony formation of hepatoma cells was inhibited by silencing SNHG17 but was promoted by overexpressing SNHG17. Representative images for Figure 4C (A) and 4D (B). Scale bar, 25 μm.

**Supplementary Fig. S7. The immunoprecipitation efficiency of the antibody against LRPPRC.** Lysates from HepG2 cells were incubated with anti-LRPPRC antibody or isotype-matched control IgG and then subjected to Western blotting. GAPDH, negative control.

**Supplementary Fig. S8.** **SNHG17 and LRPPRC do not affect each other’s subcellular localization and expression level.** (A) Silencing SNHG17 had no impact on the protein level of LRPPRC. NC- or siSNHG17-transfectants were subjected to Western blotting. (B) SNHG17 knockdown did not affect the subcellular localization of LRPPRC protein. The cytoplasm and nuclei of NC- or siSNHG17-transfectants were fractionated and then subjected to Western blotting. GAPDH and lamin A/C were used as markers for cytoplasmic and nuclear proteins, respectively. (C) LRPPRC knockdown did not affect the expression of SNHG17. NC- or siLRPPRC-transfectants were subjected to qPCR. (D) LRPPRC knockdown did not affect the subcellular localization of SNHG17. The cytoplasm and nuclei of NC- or siLRPPRC-transfectants were fractionated and then subjected to qPCR analysis. For (C-D), error bars represent mean ± SEM from three independent experiments. *P* values were assessed by unpaired Student’s *t*-test. ns, not significant.

**Supplementary Fig. S9. SNHG17 or LRPPRC may regulate the protein level of c-Myc indirectly.** (A) RNA pulldown assay did not detect an interaction between SNHG17 and c-Myc protein. The proteins from HepG2 cells were pulled down with biotin-labeled SNHG17 or SNHG17-AS and then subjected to Western blotting using anti-c-Myc antibody. (B) RNA immunoprecipitation assay did not detect an interaction between c-Myc mRNA and LRPPRC protein. The LRPPRC-RNA complex in HepG2 cells was immunoprecipitated by anti-LRPPRC antibody or isotype-matched control IgG, and the amount of c-Myc mRNA in the precipitate was analyzed by qPCR. (C) Co-IP assay did not reveal an association between LRPPRC and c-Myc protein. HepG2 lysate was precipitated with an antibody against c-Myc or isotype-matched control IgG and then subjected to Western blotting using anti-LRPPRC antibody. (D) LRPPRC knockdown did not affect the mRNA level of c-Myc. NC- or siLRPPRC-transfectants were subjected to qPCR analysis. Error bars represent mean ± SEM from three independent experiments. *P* values were assessed by unpaired Student’s *t*-test. ns, not significant.

**Supplementary Fig. S10.** **Silencing LRPPRC attenuates the roles of SNHG17 in promoting cell proliferation.** (A-B) Silencing LRPPRC attenuated the roles of SNHG17 in increasing DNA-replicating cells and cell growth. SF cells stably expressing SNHG17 and the control cells (Ctrl) were transfected with NC or siLRPPRC, then subjected to EdU incorporation assays (A) or cell counting (B). Error bars represent mean ± SEM from three independent experiments. *P* values were assessed by unpaired Student’s *t*-test *, *P* < 0.05; **, *P* < 0.01; ***, *P* < 0.001; ns, not significant.

**Supplementary Fig. S11.** **LRPPRC is up-regulated in various malignancies.** (A) Pan-cancer analysis of LRPPRC expression based on TCGA data. The levels of LRPPRC in paired tumor (T) and non-tumor (N) tissues from 10 different cancer types are presented. LIHC, liver hepatocellular carcinoma; BLCA, bladder urothelial carcinoma; BRCA, breast invasive carcinoma; HNSC, head and neck squamous cell carcinoma; KIRC, kidney renal clear cell carcinoma; KIRP, kidney renal papillary cell carcinoma; LUAD, lung adenocarcinoma; LUSC, lung squamous cell carcinoma; PRAD, prostate adenocarcinoma; STAD, stomach adenocarcinoma. (B) Kaplan-Meier analysis revealed a significant association between higher LRPPRC level and shorter survival of HCC patients. The median LRPPRC level in all 364 HCC tissues, derived from TCGA, was chosen as the cut-off value to separate the high-LRPPRC group (n = 182) from the low-SNHG17 group (n = 182). *P* values were assessed by paired Student’s *t*-test (A), or log-rank test (B). *, *P* < 0.05; ***, *P* < 0.001; ns, not significant.

**Supplementary Fig. S12.** **Autophagy inhibitor has no impact on the role of siLRPPRC in decreasing c-Myc level.** NC- or siLRPPRC-transfected cells were treated with 20 ug/ml chloroquine (CQ) for 36 hours before Western blotting.

**Supplementary Tables**

**Table S1**. Sequences of DNA and RNA oligonucleotides

| **Name** | **Sense Strand/Sense Primer (5' - 3')** | **Antisense Strand/Antisense Primer (5' - 3')** |
| --- | --- | --- |
| **siRNA duplexes** |  |  |
| siSNHG17-1 | GGAAUGACUUUAAUAACCAdTdT | UGGUUAUUAAAGUCAUUCCdAdG |
| siSNHG17-2 | CCAUGGAGUUGGUGAUCUGdTdT | CAGAUCACCAACUCCAUGGdTdC |
| siLRPPRC-1 | GAUGAGAGAUGCCGGAAUUdTdT | AAUUCCGGCAUCUCUCAUCdCdT |
| siLRPPRC-2 | UUAGGACAUCUCUUAUAGGdTdT | CCUAUAAGAGAUGUCCUAAdTdT |
| sic-Myc-1 | GAGAAUGUCAAGAGGCGAAdTdT | UUCGCCUCUUGACAUUCUCdCdT |
| sic-Myc-2 | ACGGAACUCUUGUGCGUAAdTdT | UUACGCACAAGAGUUCCGUdAdG |
| NC | UUCUCCGAACGUGUCACGUdTdT | ACGUGACACGUUCGGAGAAdTdT |
| **Primers for qPCR** | | |
| SNHG17 | TGGGAGAGCATGGATCCTGA | GCAGCTCAGCCTCTTCTTGA |
| LRPPRC | AGCCCAGCCAGGCTGTATG | CTACCACCTAGGCCACCTGAG |
| c-Myc | TCTTCCCCTACCCTCTCAAC | TCCAGACTCTGACCTTTTGC |
| MALAT1 | TCGTTTGCCTCAGACAGGTA | GGAAGGGGTCAGGAGAAAGTG |
| cyclin A2 | TTATTGCTGGAGCTGCCTTT | CTCTGGTGGGTTGAGGAGAG |
| CDC6 | AAGCTGTCTCGGGCATTGAA | TGCCTTGCTTTGGTGGAGAA |

**Table S1. Sequences of DNA and RNA oligonucleotides (Continued)**

| **Name** | **Sense Strand/Sense Primer (5' - 3')** | **Antisense Strand/Antisense Primer (5' - 3')** | |
| --- | --- | --- | --- |
| MCM3 | AGTTCGTCCCAAAGTCGTCC | CCTGGATGGTGATGGTCTGG | |
| TK1 | TGCTCAGTACAAGTGCCTGG | TCGTCGATGCCTATGACAGC | |
| DHFR | CACAAGGAGCTCATTTTCTTTCC | AGTTTAAGATGGCCTGGGTGA | |
| GAPDH | GAGTCAACGGATTTGGTCGT | GACAAGCTTCCCGTTCTCAG | |
| U6 | CTCGCTTCGGCAGCACA | AACGCTTCACGAATTTGCGA | |
| **Primers for RACE** | |  | |
| **3’RACE** |  |  | |
| 3’RACE-adaptor-RT | ATGGCAGCAAGGTGATCACTAAAGTGATATCCTTTTTTTTTTTTTTTTVN | |  |
| 3’RACE-adaptor | | ATGGCAGCAAGGTGATCACTAAA | |
| Nest PCR-inner | GATTGTCAGCTGACCTCTGTCCT |  | |
| Nest PCR-outer | GTGTGAGAGGCGTCCTCTGG |  | |
| **5’RACE** |  |  | |
| Nest PCR-inner | AGGACAGAGGTCAGCTGACAATC |  | |
| Nest PCR-outer | CCAGAGGACGCCTCTCACAC |  | |
| 5’RACE-adaptor-primer-inner | | GCTCTGGTGACGCTTCATGTGGTAGCCT | |

**Table S1. Sequences of DNA and RNA oligonucleotides (Continued)**

| **Name** | | **Sense Strand/Sense Primer (5' - 3')** | **Antisense Strand/Antisense Primer (5' - 3')** | |  |
| --- | --- | --- | --- | --- | --- |
| 5’RACE-adaptor-primer-outer | | | TCTGGCTCTTGGCATCAACTCACCACG | |  |
| **Primers for cloning into pcDNA3.0 (restriction enzyme sites are underlined)** | | | | |  |
| **SNHG17** | |  |  | |  |
| Full length | | ATCGAATTCGTATTTCCGCCGGCGCGAA | ATCTCTAGAGCAATTCTGTAAGGTTTATTGAATGGGT | |  |
| 1-588 nt | | ATCGAATTCGTATTTCCGCCGGCGCGAA | ATCTCTAGAGCCCCTCTCGTGCCCACAAAC | |  |
| 1-284 nt | | ATCGAATTCGTATTTCCGCCGGCGCGAA | ATCTCTAGACGGACAGTGGAAGCCCCTCAG | |  |
| 1-150 nt | | ATCGAATTCGTATTTCCGCCGGCGCGAA | ATCTCTAGACCTGACAGACAGCGTGGGAAAAATC | |  |
| 134-284 nt | | ATCGAATTCCCACGCTGTCTGTCAGGTCTCC | ATCTCTAGACCTGACAGACAGCGTGGGAAAAATC | |  |
| 284-862 nt | | ATCGAATTCCTGAGGGGCTTCCACTGTCCG | ATCTCTAGACCTGACAGACAGCGTGGGAAAAATC | |  |
| antisense SNHG17 | | ATCGCGGCCGCGTATTTCCGCCGGCGCGA | ATCGAATTCGCAATTCTGTAAGGTTTATTGAATGGGT | |  |
| **Primer for cloning into pCDH-CMV-MCS-EF1-CopGFP-T2A-Puro (restriction enzyme sites are underlined)** | | | | |  |
| SNHG17 | | ATCTCTAGAGTATTTCCGCCGGCGCGAA | ATCGAATTCGCAATTCTGTAAGGTTTATTGAATGGGT | |  |
| SNHG17-Δcore | | ATCTCTAGAGATTTTTCCCACGCTGTCTGTCAGG | ATCGAATTCGCAATTCTGTAAGGTTTATTGAATGGGT | |  |
| SNHG17-ORF1 | | TCATCTAGAGTATTTCCGCCGGCGCGAAAC | TCAGAATTCTCACTTATCGTCGTCATCCTTGTAATCGCCTCTTCTTGAAGACACGTCACCTGG | |  |
| SNHG17-ORF2 | TCATCTAGAGTATTTCCGCCGGCGCGAAAC | | | TCAGAATTCTCACTTATCGTCGTCATCCTTGTAATCCTCTCCATTCTCTGCCCCTCTCGTG | |

**Table S1. Sequences of DNA and RNA oligonucleotides (Continued)**

| **Name** | **Sense Strand/Sense Primer (5' - 3')** | **Antisense Strand/Antisense Primer (5' - 3')** |
| --- | --- | --- |
| SNHG17-ORF3 | TCATCTAGAGTATTTCCGCCGGCGCGAAAC | TCAGAATTCTCACTTATCGTCGTCATCCTTGTAATCTTCCAGGCATGGACAGAGGGATGC |
| **Primer for cloning into pGEX-6P-1 (restriction enzyme sites are underlined)** | | |
| **LRPPRC** |  |  |
| Full length | TACGGATCCGCAGCCCTGCTGAGATCCG | TACGCGGCCGCTCAAGAAGAGTTTTCCCTCAATTTT |
| 1-1032 nt | TACGGATCCGCAGCCCTGCTGAGATCCG | TACGCGGCCGCTCAAAGTAAAATGAGGTTCATTGCATCTG |
| 1033-2064 nt | TACGGATCCTTAGTCACTGAAAAATTGGAAGATGT | TACGCGGCCGCTCACACTAATATGAGTTGCTTTAGGACATC |
| 2065-3102 nt | TACGGATCCAAGCAACTCATATTAGTGCTTTGTTC | TACGCGGCCGCTCAATCAGGTTCTGTGGTTGAGGCT |
| 3103-4107 nt | TACGGATCCTTCCAGAAAGATATATTGATTGCCT | TACGCGGCCGCTCAAGAAGAGTTTTCCCTCAATTTT |
