# Supplementary figures and images for: LncRNA SNHG17 interacts with LRPPRC to stabilize c-Myc protein and promote G1/S transition and cell proliferation
Source: Cell Death Dis. 2021 Oct 20;12(11):970. doi: 10.1038/s41419-021-04238-x (PMC8528917; doi:10.1038/s41419-021-04238-x)

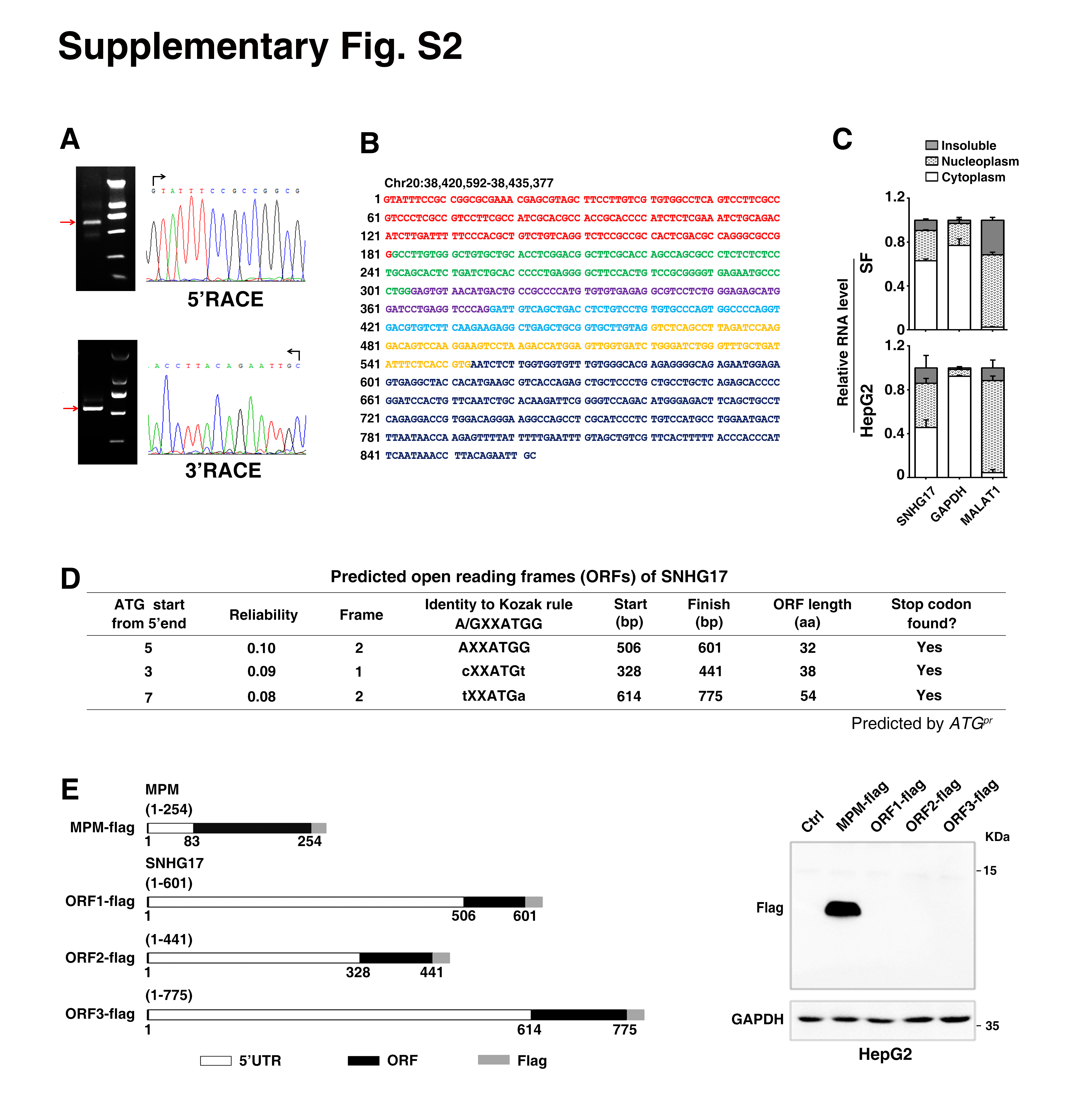

Supplement: Supplementary file 3 — Supplementary Figure S2 [file 41419_2021_4238_MOESM3_ESM.tif]

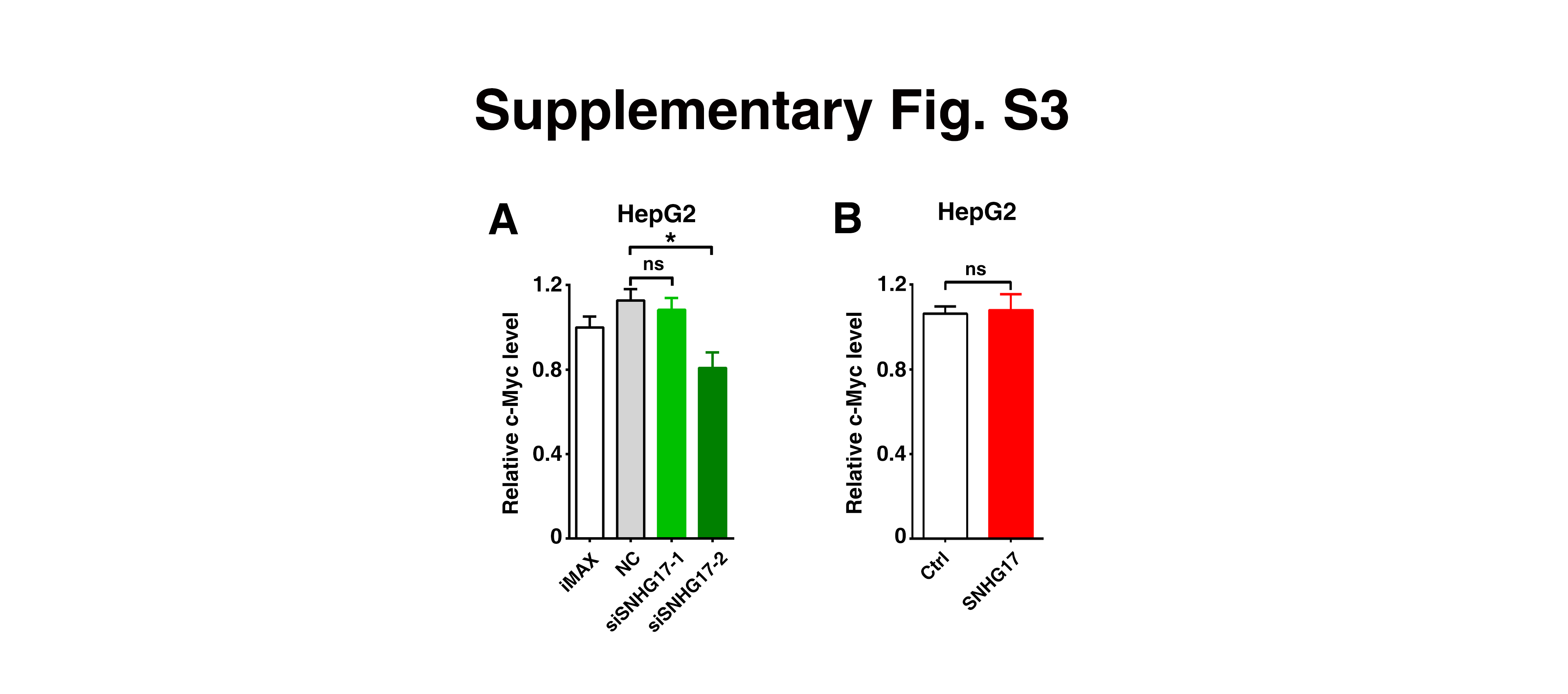

Supplement: Supplementary file 4 — Supplementary Figure S3 [file 41419_2021_4238_MOESM4_ESM.tif]

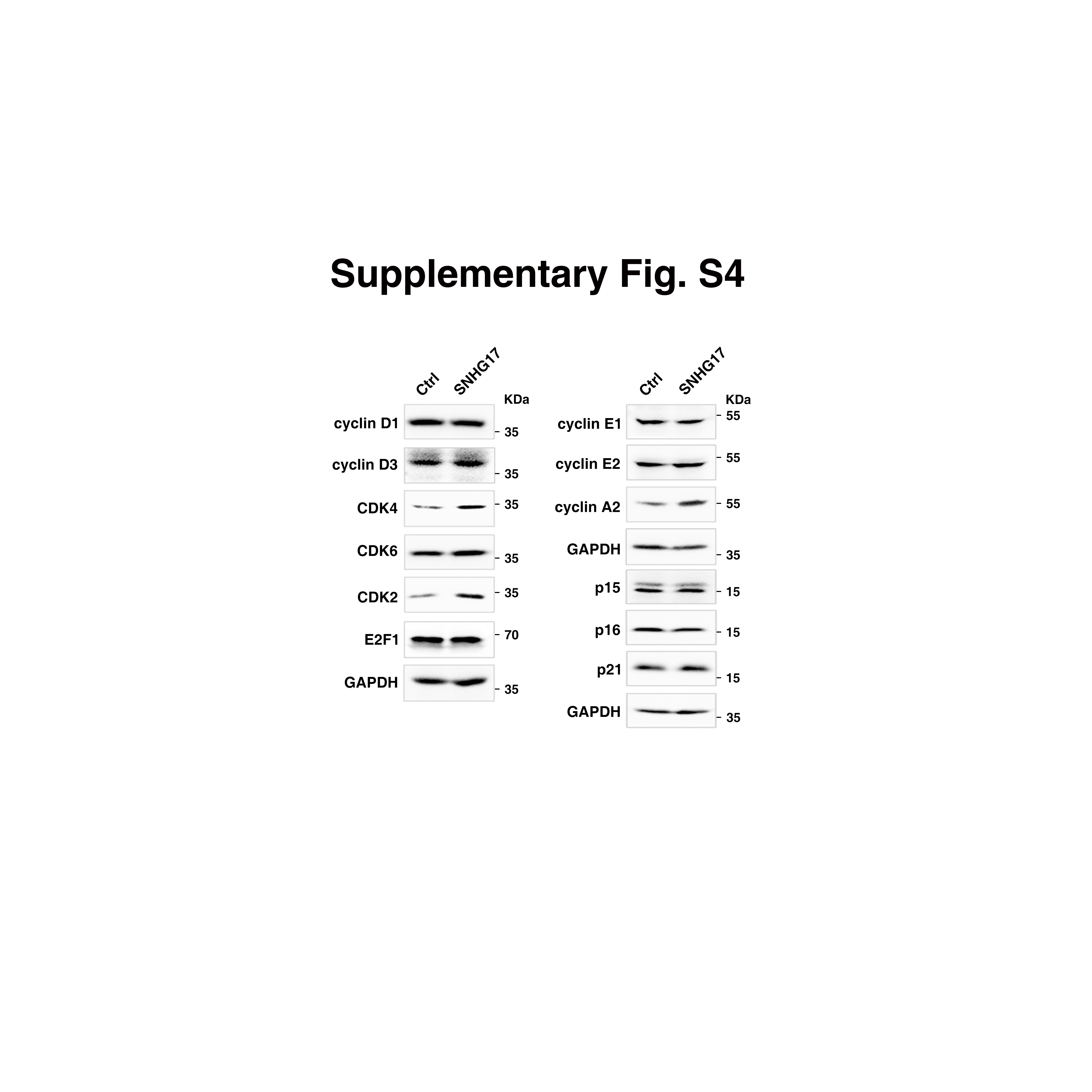

Supplement: Supplementary file 5 — Supplementary Figure S4 [file 41419_2021_4238_MOESM5_ESM.tif]

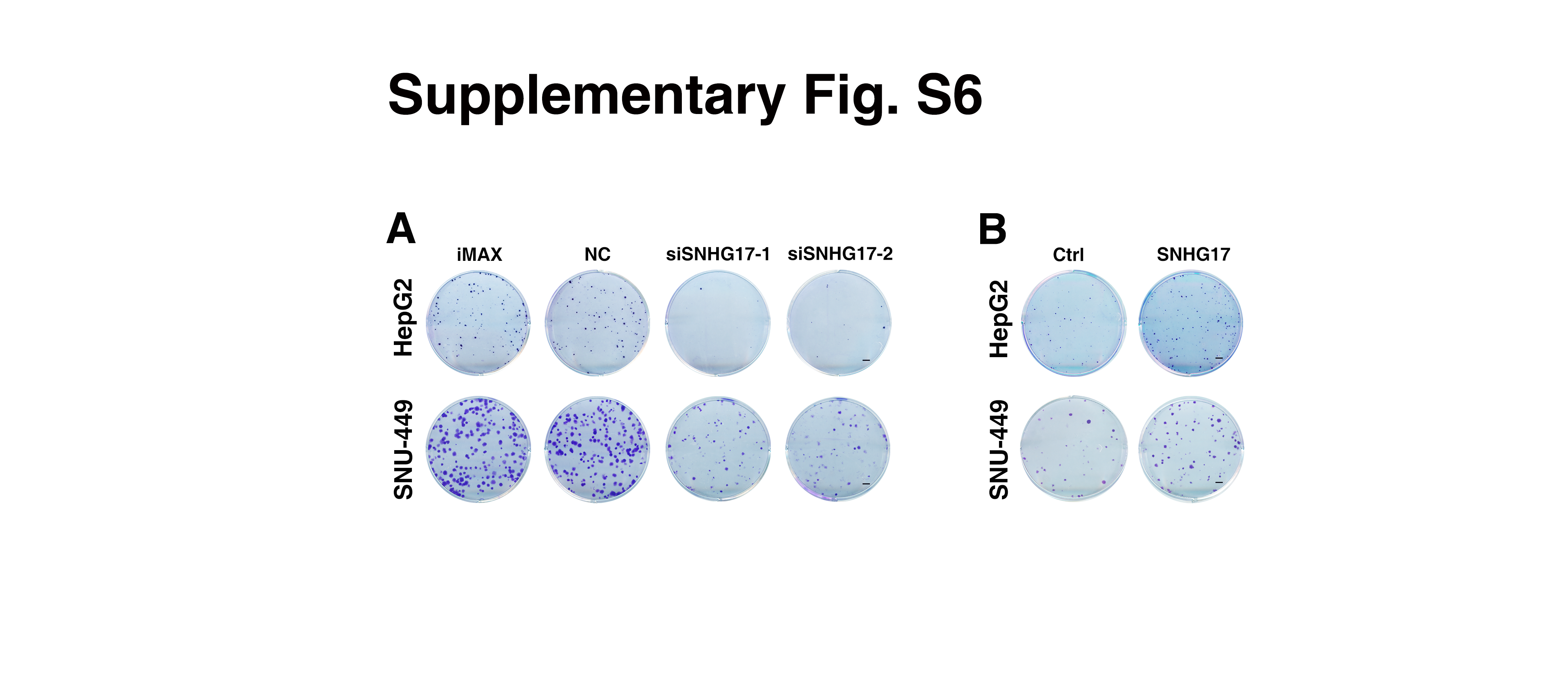

Supplement: Supplementary file 7 — Supplementary Figure S6 [file 41419_2021_4238_MOESM7_ESM.tif]

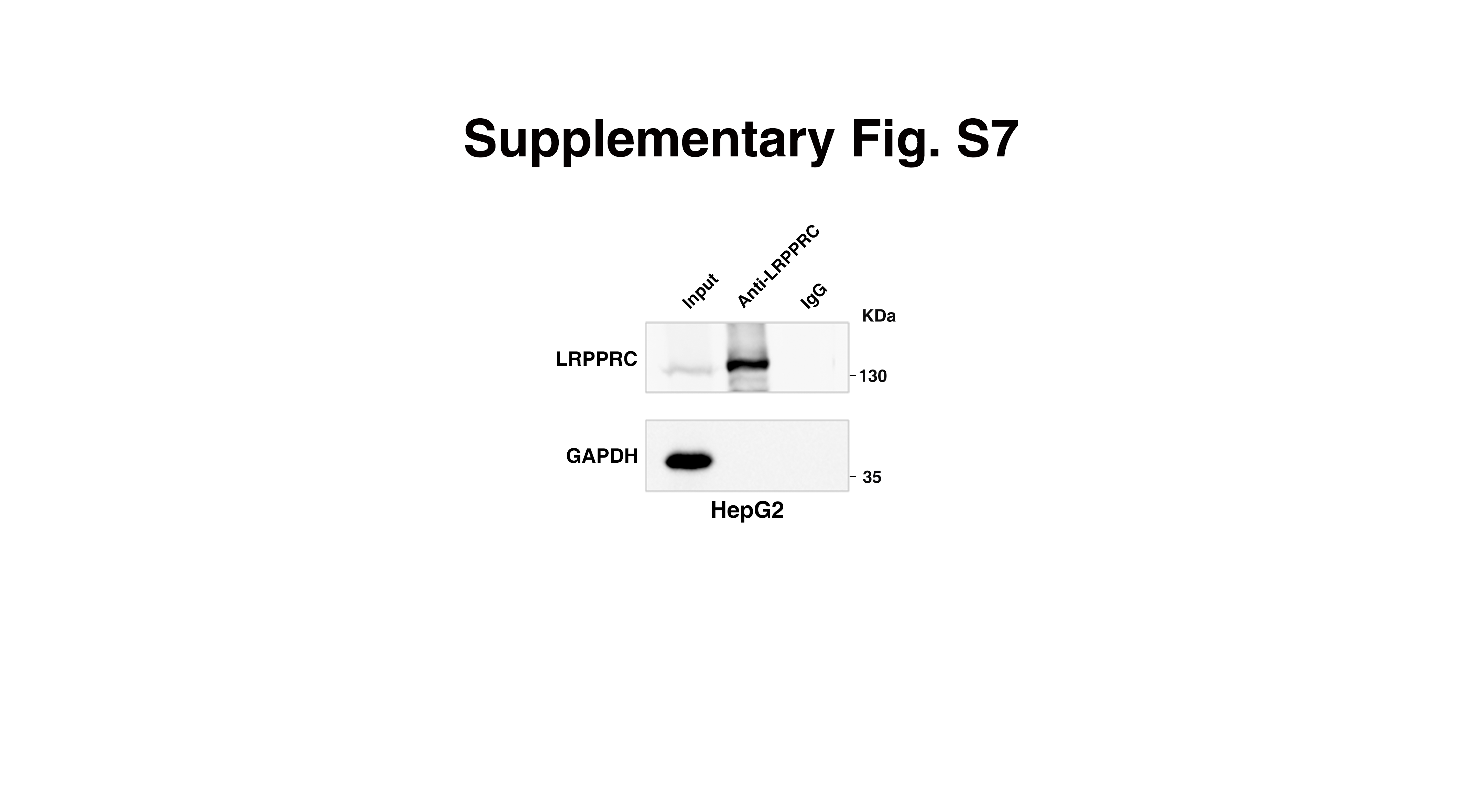

Supplement: Supplementary file 8 — Supplementary Figure S7 [file 41419_2021_4238_MOESM8_ESM.tif]

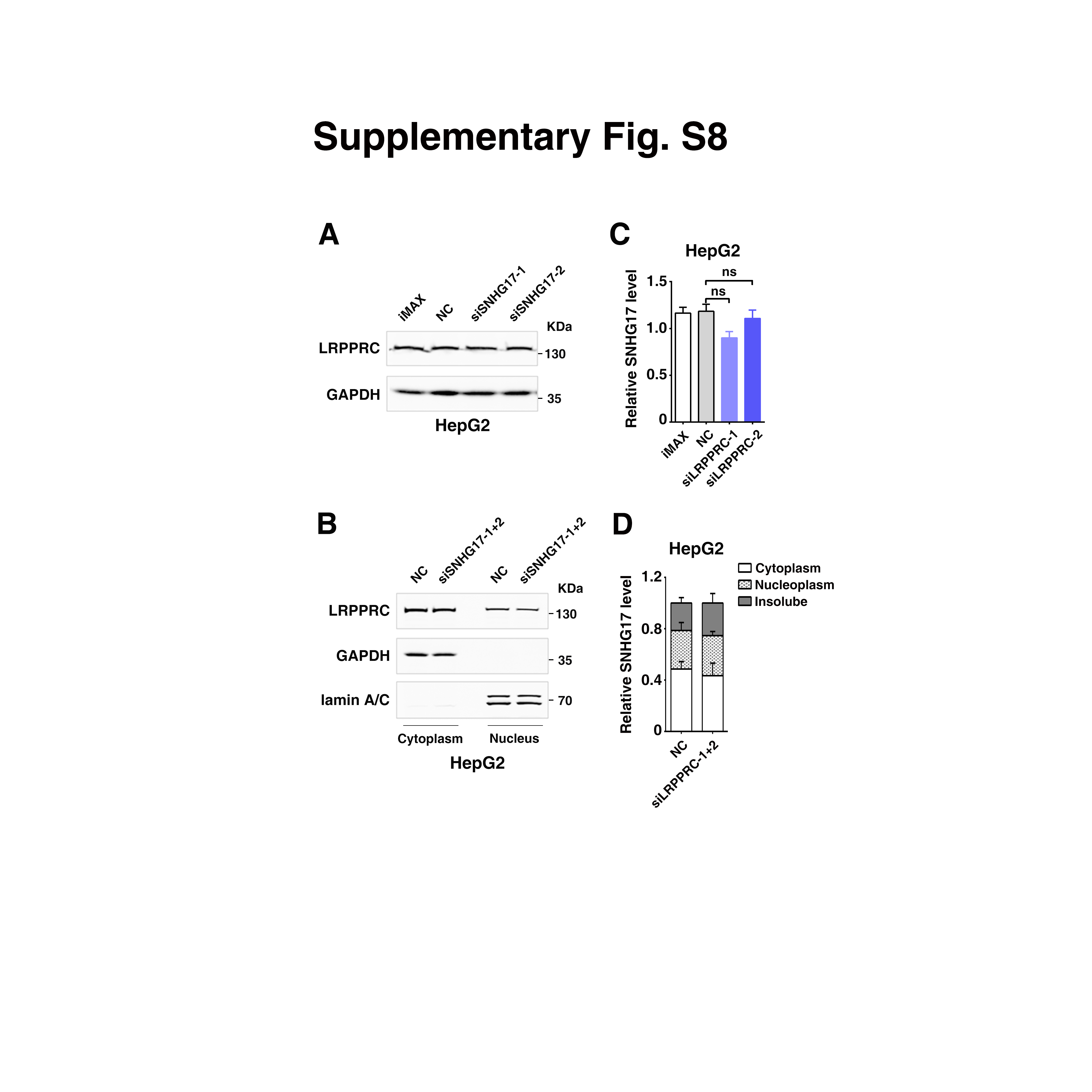

Supplement: Supplementary file 9 — Supplementary Figure S8 [file 41419_2021_4238_MOESM9_ESM.tif]

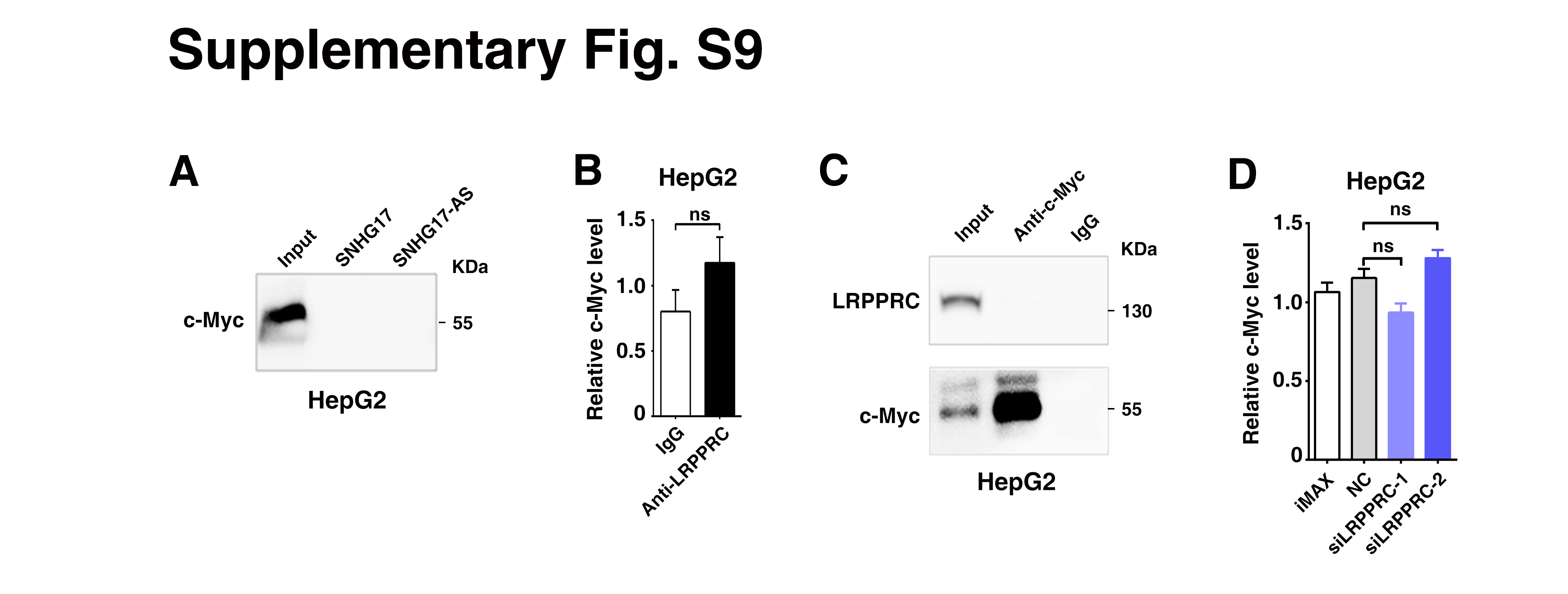

Supplement: Supplementary file 10 — Supplementary Figure S9 [file 41419_2021_4238_MOESM10_ESM.tif]

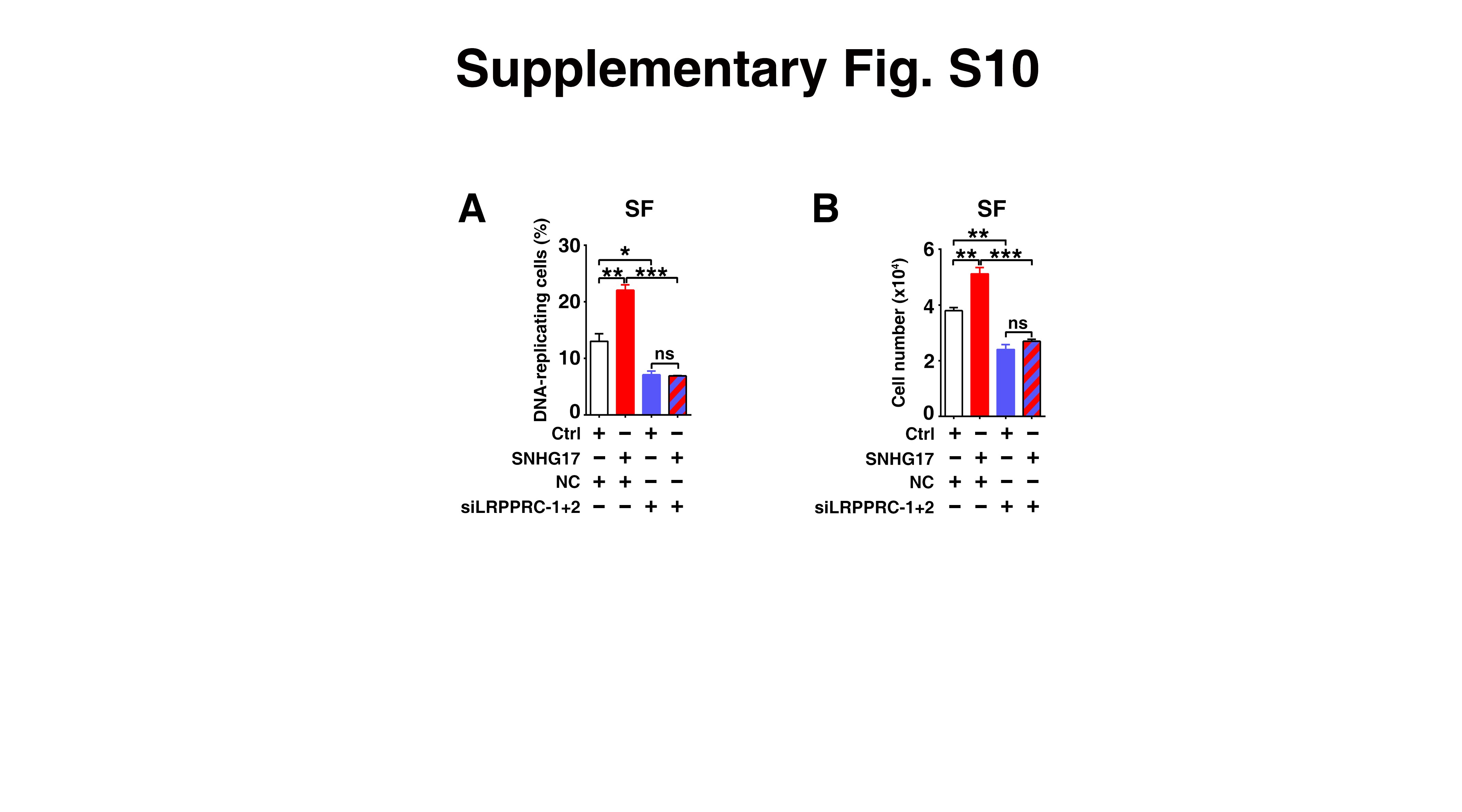

Supplement: Supplementary file 11 — Supplementary Figure S10 [file 41419_2021_4238_MOESM11_ESM.tif]

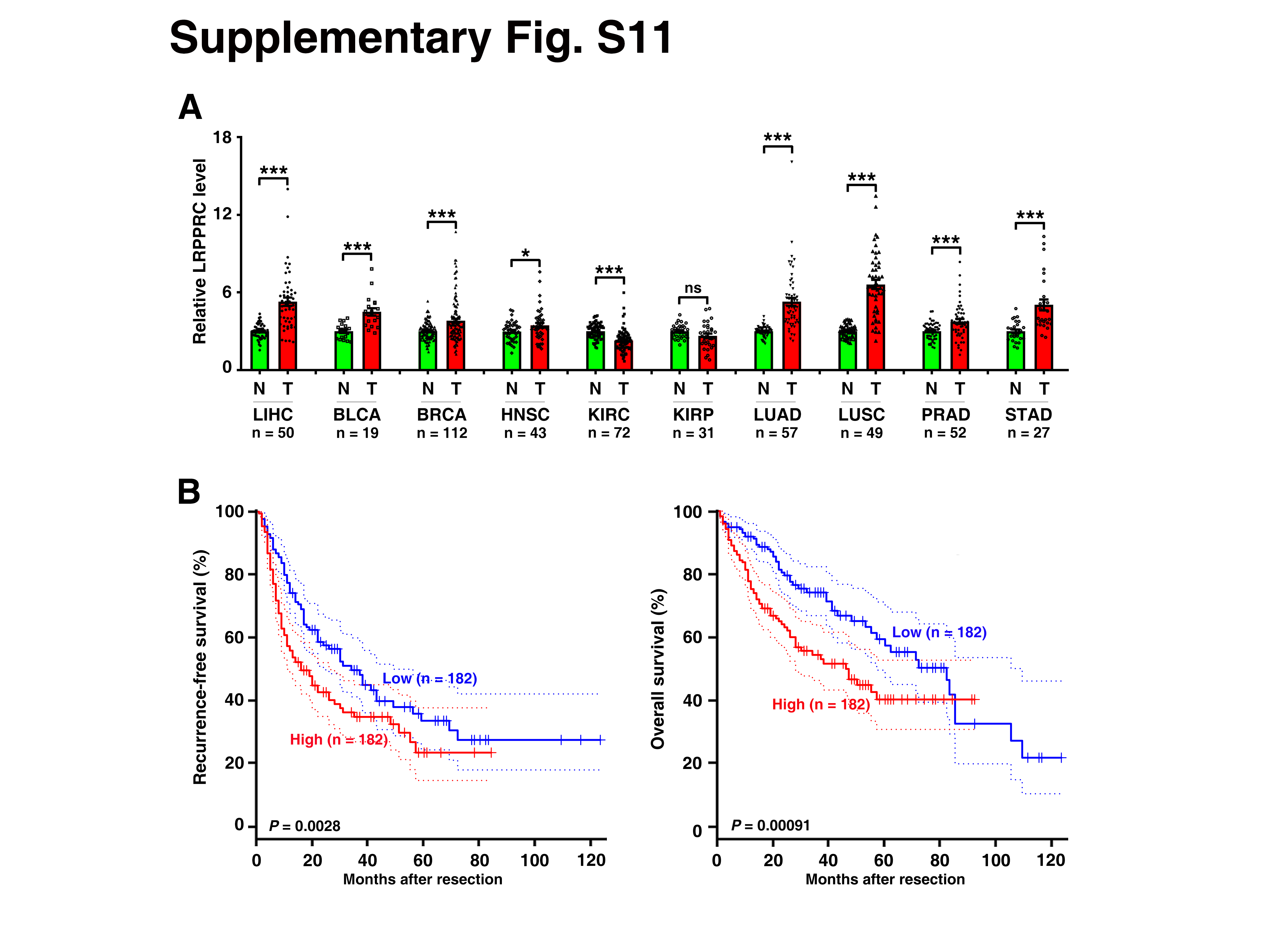

Supplement: Supplementary file 12 — Supplementary Figure S11 [file 41419_2021_4238_MOESM12_ESM.tif]

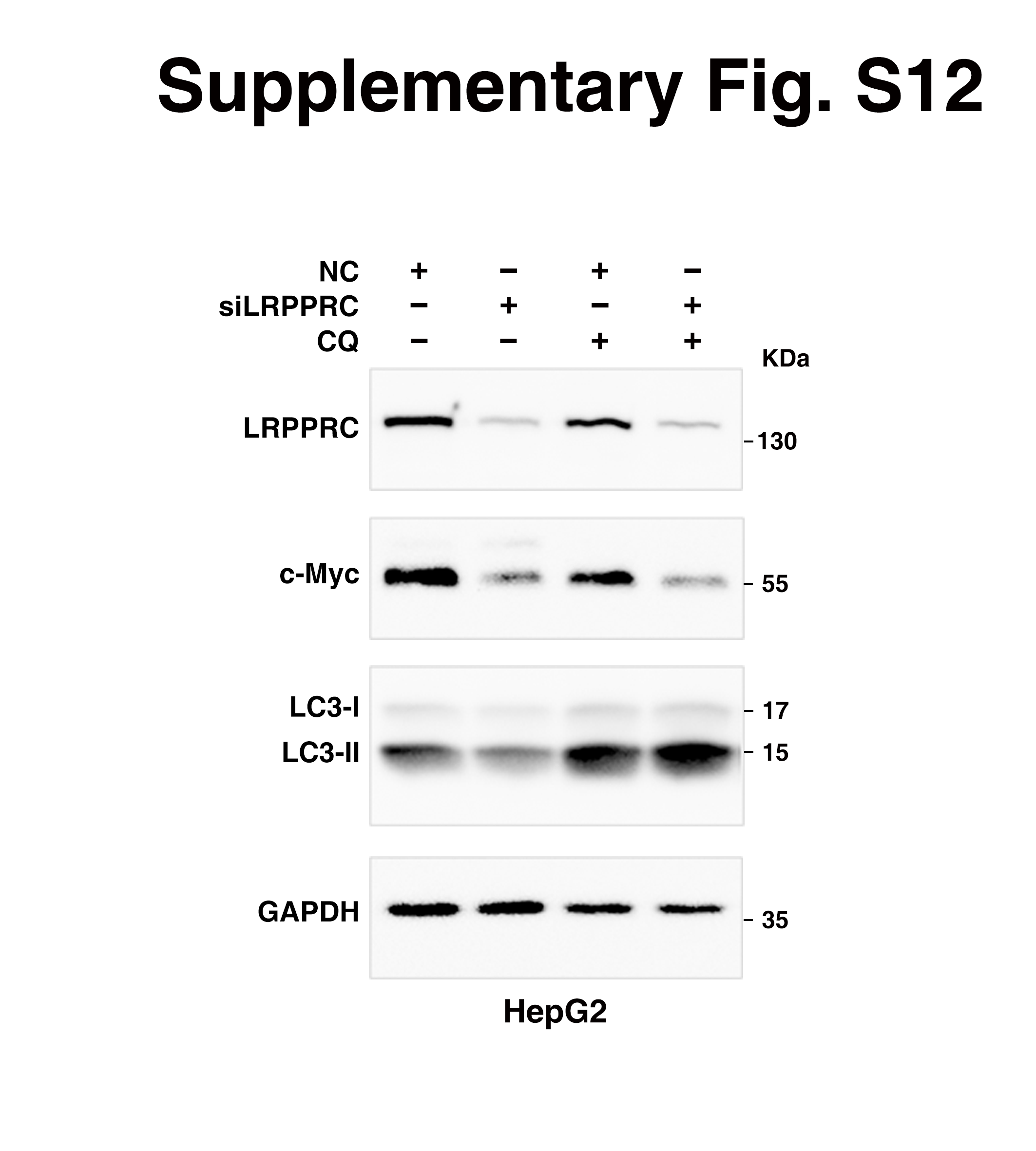

Supplement: Supplementary file 13 — Supplementary Figure S12 [file 41419_2021_4238_MOESM13_ESM.tif]
